# Insights for precision oncology from the integration of genomic and clinical data of 13,880 tumors from the 100,000 Genomes Cancer Programme

**DOI:** 10.1038/s41591-023-02682-0

**Published:** 2024-01-11

**Authors:** Alona Sosinsky, John Ambrose, William Cross, Clare Turnbull, Shirley Henderson, Louise Jones, Angela Hamblin, Prabhu Arumugam, Georgia Chan, Daniel Chubb, Boris Noyvert, Jonathan Mitchell, Susan Walker, Katy Bowman, Dorota Pasko, Marianna Buongermino Pereira, Nadezda Volkova, Antonio Rueda-Martin, Daniel Perez-Gil, Javier Lopez, John Pullinger, Afshan Siddiq, Tala Zainy, Tasnim Choudhury, Olena Yavorska, Tom Fowler, David Bentley, Clare Kingsley, Sandra Hing, Zandra Deans, Augusto Rendon, Sue Hill, Mark Caulfield, Nirupa Murugaesu

**Affiliations:** 1 Genomics England, London, UK; 2 School of Life Sciences, University of Westminster, London, UK; 3 Institute of Cancer Research, London, UK; 4 Genomics Unit, NHS England, London, UK; 5 Barts Cancer Institute, Queen Mary University of London, London, UK; 6 Oxford University Hospitals NHS Foundation Trust, Churchill Hospital, Oxford, UK; 7 Institute of Cancer and Genomic Sciences, University of Birmingham, Birmingham, UK; 8 William Harvey Research Institute and the Barts Cancer Institute, Queen Mary University of London, London, UK; 9 Illumina Cambridge, Cambridge, UK; 10 Guy’s & St Thomas’ NHS Foundation Trust, London, UK

**Keywords:** Genetic testing, Cancer genomics

## Abstract

The Cancer Programme of the 100,000 Genomes Project was an initiative to provide whole-genome sequencing (WGS) for patients with cancer, evaluating opportunities for precision cancer care within the UK National Healthcare System (NHS). Genomics England, alongside NHS England, analyzed WGS data from 13,880 solid tumors spanning 33 cancer types, integrating genomic data with real-world treatment and outcome data, within a secure Research Environment. Incidence of somatic mutations in genes recommended for standard-of-care testing varied across cancer types. For instance, in glioblastoma multiforme, small variants were present in 94% of cases and copy number aberrations in at least one gene in 58% of cases, while sarcoma demonstrated the highest occurrence of actionable structural variants (13%). Homologous recombination deficiency was identified in 40% of high-grade serous ovarian cancer cases with 30% linked to pathogenic germline variants, highlighting the value of combined somatic and germline analysis. The linkage of WGS and longitudinal life course clinical data allowed the assessment of treatment outcomes for patients stratified according to pangenomic markers. Our findings demonstrate the utility of linking genomic and real-world clinical data to enable survival analysis to identify cancer genes that affect prognosis and advance our understanding of how cancer genomics impacts patient outcomes.

## Abstract (web summary)

The first report from the Cancer Programme of the 100,000 Genomes Project presents whole-genome sequencing profiles of 13,880 solid tumors spanning 33 cancer types, combined with real-world clinical data, providing insights for precision oncology.

## Main

**Fig. 1:**
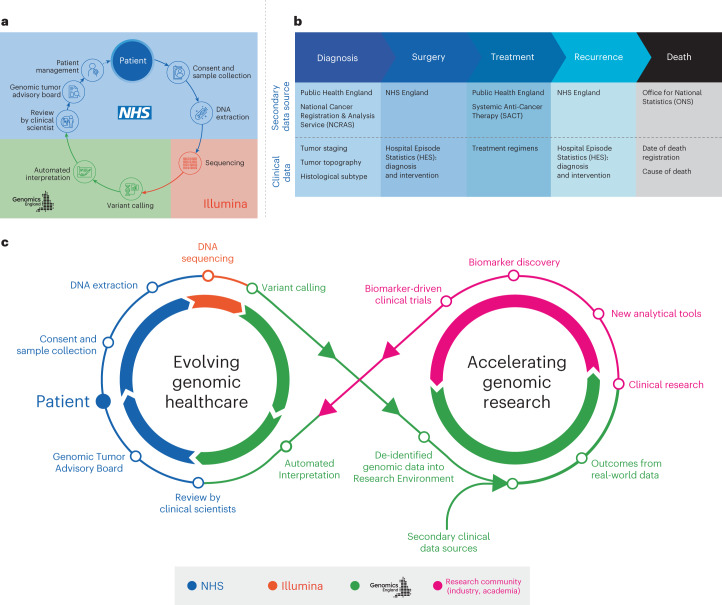
Overview of the 100,000 Genomes Cancer Programme. **a**, Journey of the patient’s genome. Patients provided written informed consent for paired tumor and normal (germline) WGS analysis. DNA was extracted from tumor and normal (blood) samples using standardized protocols and samples were submitted for WGS, which was performed on an Illumina sequencer. An automated pipeline was constructed for sequence quality control, alignment, variant calling and interpretation, with results returned to the 13 NHS Genomic Medicine Centers for review in regional GTABs. **b**, Linked genomic and real-world clinical datasets. In the 100,000 Genomes Project, participants are followed over their life course using electronic health records (all hospital episodes, cancer registration entries, systemic anticancer therapies and cause of death). **c**, Infinity loop representing the link between healthcare and research in genomics.

Over the last decade, UK cancer incidence has increased by approximately 4% (ref. ^1^), driving the need for molecular cancer testing, including germline testing of cancer predisposition genes and pharmacogenomic markers^2^. The 100,000 Genomes Project, a transformational UK Government initiative conducted within the National Health Service (NHS) in England, aimed to establish standardized high-throughput whole-genome sequencing (WGS) for patients with cancer and rare diseases via an automated, International Organization for Standardization-accredited bioinformatics pipeline (providing clinically accredited variant calling and variant prioritization)^3^. The role of WGS at scale for patients with cancer in the NHS was evaluated within the Cancer Programme of the 100,000 Genomes Project (Fig. 1a). Participants gave written informed consent for their genomic data to be linked to anonymized longitudinal health records and shared with researchers in a secure Research Environment (www.genomicsengland.co.uk/research/research-environment) to drive forward our knowledge across different cancers^4^. The data generated were then used to establish a national molecular data platform (National Genomic Research Library) with secure links to longitudinal real-world data in the Research Environment (Fig. 1b). The national clinical datasets include the National Cancer Registration and Analysis Service (NCRAS) dataset consisting of cancer registration data and the Systemic Anti-Cancer Therapy (SACT) dataset, as well as subsequent cancer episodes, including Hospital Episode Statistics (HES) and mortality data from the Office for National Statistics (ONS)^5^ (Fig. 1b). This approach enables genomic research and discovery to be fed back into genomic healthcare (Fig. 1c).

A longer-term objective was to accelerate the delivery of molecular testing, including WGS, in NHS clinical cancer care^6^. Building on evolving knowledge from the 100,000 Genomes Project and the existing molecular testing provision within the NHS, the NHS Genomic Medicine Service (GMS) was launched in October 2018 to deliver genomic testing, clinical care and interpretation for rare diseases and cancer across England, using a standardized National Genomic Test Directory^7^, including targeted large gene panels and WGS, to enable equitable access and comprehensive genomic testing. The National Genomic Test Directory aims to provide consistency of test methodologies, gene targets and eligibility criteria across clinical indications via a consolidated network of seven NHS England (NHSE) Regional Genomic Laboratory Hubs^8^. It specifies the genomic tests that are commissioned and thereby funded by the NHS in England as part of gold standard molecular profiling in different cancer clinical indications and provides opportunities for patients to participate in research^9^.

Large-scale sequencing studies such as the International Cancer Genome Consortium (ICGC) and The Cancer Genome Atlas (TCGA) have extensively cataloged the spectra of somatic mutations across cancer types from a retrospective cohort of 2,658 primary tumor samples^10^. More recent initiatives, such as The Hartwig Medical Foundation reported clinically relevant findings for 4,784 metastatic adult solid tumor samples^11^ and supported recruitment to the Drug Rediscovery Protocol (DRUP) trial^12^. These initiatives represent, to date, the two largest WGS cohorts available for research. In this article, we present our analysis of WGS data from 13,880 solid tumors, focused on clinically actionable genes and pangenomic markers, linked to real-world longitudinal, life course clinical, treatment and long-term survival data to highlight the learnings from the Cancer Programme and the implications for current clinical care.

## Results

### Cohort demographics

**Fig. 2:**
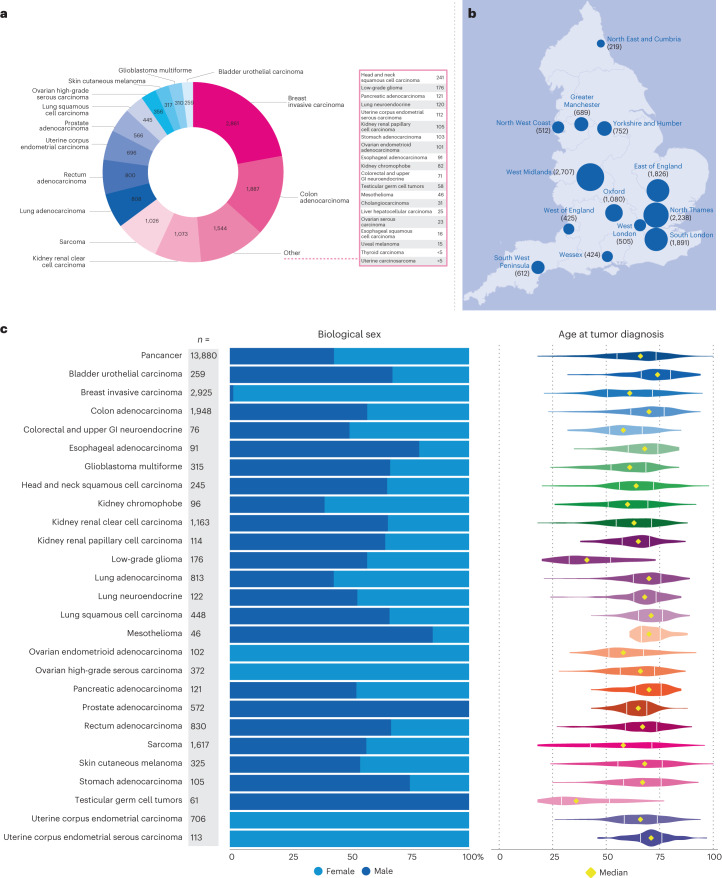
Overview of the 100,000 Genomes Cancer Programme cohort demographics. **a**, Distribution of 12,948 cases represented by 33 tumor types (cases with more than one sample per tumor were only counted once). **b**, Thirteen NHS GMCs recruited patients diagnosed with cancer across England. The area of the pie chart is proportional to the number of patients recruited; the total number of participants recruited per GMC is indicated in parentheses. Map source: Office for National Statistics licensed under the Open Government Licence v.3.0. **c**, Breakdown of biological sex and age at diagnosis according to disease. The age plot shows the interquartile range (IQR) and median values.

We sequenced 16,358 tumor-normal sample pairs from 15,241 patients diagnosed with cancer within the NHS who were recruited to the Cancer Programme of the 100,000 Genomes Project between 2015 and 2019, with almost half of the patients being recruited in 2018 and the remainder in this Project being recruited through the Rare Disease arm. Our integrative whole-genome analysis (WGA) covered 33 tumor types (Fig. 2a) of 13,880 tumor samples, consisting of 13,311 fresh-frozen (95.9%) and 569 formalin-fixed paraffin-embedded tumor samples (4.1%). Matched normal (germline) samples included 13,493 (99.1%) blood-derived, 100 (0.7%) from normal tissue and 23 (0.2%) from saliva samples. Tumor samples were sequenced to 100× coverage and normal samples to 30× to ensure high sensitivity of variant calling (Methods) in clinical settings (compared with 60× and 38× in the TCGA cohort). Genomes from hematological tumors (*n* = 841), pediatric cancers (*n* = 333), carcinomas of unknown primary (*n* = 98) and tumors that were not linked to external datasets (*n* = 1,206) were excluded from this analysis. The diagnosis submitted at sample collection was confirmed by linking genomics data with the NCRAS and HES datasets. Tumor types with more than 1,000 sequenced tumor genomes included breast invasive carcinoma (*n* = 2925), colon adenocarcinoma (*n* = 1948), sarcoma (*n* = 1617) and kidney renal clear cell carcinoma (*n* = 1163). Figure 2b illustrates recruitment across 13 NHS GMCs (comprising over 80 hospital trusts) in England. The distribution of biological sex and age across tumor types is shown in Fig. 2c. Early onset (median age less than 50 years) was observed for low-grade glioma and testicular germ cell tumors in agreement with incidence statistics^13^.

**Fig. 3:**
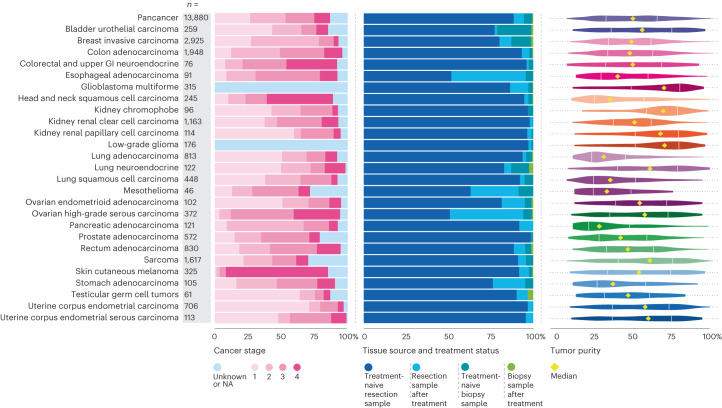
Overview of the sample characteristics for the 100,000 Genomes Cancer Programme cohort. Breakdown according to the stage of the disease (left) (NA, not available or not applicable in the context of glioblastoma multiforme and low-grade glioma), type of sample obtained (middle) and tumor purity (right) for each tumor type; the IQR and median values are shown.

Staging information was available in the NCRAS dataset for 12,040 (86.7%) tumors. The breakdown of the different stages for the tumor types sequenced is shown in Fig. 3; 11.9% (1,645 of 13,880) of patients had stage 4 cancer (advanced metastatic disease) with samples obtained from metastatic sites including the liver, lymph nodes, lung and brain. Ovarian high-grade serous carcinoma and skin cutaneous melanoma exhibited higher prevalence of advanced (stages 3 and 4) disease, whereas invasive breast cancers had a higher prevalence of early-stage (stages 1 and 2) disease due to sampling biases in tissue ascertainment. Tumor samples mainly originated from surgical resections (94.5%, *n* = 13,120), including 93.6% treatment-naive cases and 6.4% cases after neoadjuvant treatment. Only 5.5% (*n* = 760) came from metastatic or diagnostic biopsies, with 10.9% (*n* = 83) being after treatment (Fig. 3). The tumor purity depicted in Fig. 3 highlights challenges in obtaining samples with adequate tumor content (more than 30%) in specific cancers, such as lung and pancreatic adenocarcinomas, which is consistent with previous publications^14^.

### Clinical actionability through WGS

A single test such as WGS, comprising paired tumor-normal sequencing, can facilitate the concurrent detection of somatic small variants including single-nucleotide variants (SNVs) and insertions and deletions (indels), copy number aberrations (CNAs) and structural variants (SVs), including gene fusions. In addition, germline findings, such as variants in cancer susceptibility genes and pharmacogenomic findings (variants affecting the metabolism of therapeutic agents used to treat cancers), enabled a greater yield of clinically relevant findings. The Cancer Programme delivered standardized WGA results, generated in an automated bioinformatics pipeline, returned to NHS GMC Laboratories. Potentially actionable findings were reviewed initially by clinical scientists and subsequently at multidisciplinary Molecular Tumor Boards, referred to as Genomic Tumor Advisory Boards (GTABs). Examples of WGA results are shown in the Supplementary Information; full details of the analysis and interpretation are described in the Methods, showing the utility of WGS to capture various genomic alterations of clinical relevance with a single test.

**Fig. 4:**
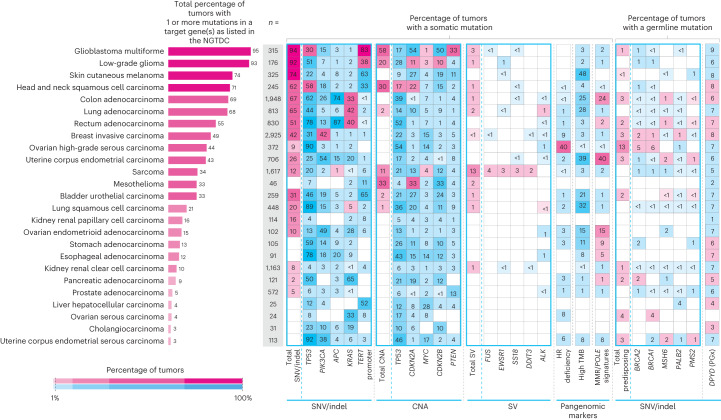
Somatic and germline alterations across common tumor types. Prevalence of different types of mutations identified using WGS in genes indicated for testing in the NGTDC. The leftmost panel indicates the total percentage of cases harboring one or more genomic alterations of clinical relevance as listed in the NGTDC (where the number of cancers sequenced is ten or more). In the subsequent panels, somatic variants (from left to right) consisting of small variants (SNVs, indels), CNAs, SVs, HRD, MMR signatures and TMB along with germline variants related to inherited cancer risk (predisposing genes) and pharmacogenomic (PGx) findings (toxicity-associated *DPYD* variants) are shown. The top five genes with the most prevalent mutation rates for each mutation type are shown (see Extended Data Fig. 1 for the full analysis). The percentage of tumors harboring a specific type of mutation in the gene(s) indicated for testing according to tumor type in the NGTDC are shown in magenta. Mutation incidence (as a percentage) in other tumor types, not currently indicated in the NGTDC, is shown in blue. Color gradation reflects the percentage of affected cases.

We analyzed aggregated data from 13,880 whole genomes in the context of the current National Genomic Test Directory for Cancer (NGTDC) v.6.0 updated on 3 April 2023 (ref. ^7^); several types of mutations relating to targets specified in the NGTDC were detected, including small variants, CNAs and fusions, along with germline variants associated with inherited cancer risk and pharmacogenomic findings (see the online Methods for details). The percentage of cases with one or more somatic mutations present in genes indicated in the NGTDC for the applicable cancer type was high, although variable (Fig. 4). For example, over 50% of tumors harbored one or more mutations found in genes indicated for testing in the NGTDC in glioblastoma multiforme, low-grade glioma, skin cutaneous melanoma, head and neck squamous cell carcinoma, colon and rectal adenocarcinoma, and lung adenocarcinoma (Fig. 4). Clinically relevant mutations were found in 20–49% of breast invasive carcinoma, ovarian high-grade serous carcinoma, uterine endometrial, sarcoma, mesothelioma, bladder urothelial carcinoma and lung squamous cell carcinoma cases, while in other cancer types such as pancreatic, prostate, esophageal and stomach adenocarcinomas, less than 20% of cases possessed mutations in genes present in the NGTDC (Fig. 4). We note that the clinical actionability of these mutations will be dependent on the individual case and clinical circumstances, such as the stage of the tumor and associated comorbidities of the participant. This highlights the need for clinical interpretation and discussion where clinically appropriate within a GTAB.

We assessed the mutations listed in the NGTDC in other cancer types for which testing of that gene or mutation is not currently indicated (Fig. 4 and in Extended Data Fig. 1a–d). These variants are denoted in blue in Fig. 4 and could indicate potentially actionable findings that may enable recruitment into clinical trials or prompt further review within a GTAB. For example, SNVs were identified in *PIK3CA* and *KRAS* across different cancer types and similarly pangenomic markers, such as homologous recombination deficiency (HRD) and tumor mutational burden (TMB), for which clinical trials may be available. As biomarker-driven trial evidence grows, NGTDC indications are expected to expand, incorporating new genes and biomarkers across several cancer types.

### Landscape of somatic small variants

The most frequently mutated gene was *TP53* (5,411 of 13,880, 39.0% of patients; Fig. 4 and online Methods). Within individual cancer types, the frequency of *TP53* mutations was variable but highest in uterine corpus endometrial serous carcinoma, ovarian high-grade serous carcinoma, lung squamous cell carcinoma, rectum adenocarcinoma, esophageal adenocarcinoma and esophageal squamous cell carcinoma (more than 70% of cases). Of the individuals with at least one *TP53* mutation, 36.2% (1,959 of 5,411) harbored one or more variant predicted to be protein-truncating or splice-altering and 65.5% (3,544 of 5,411) carried one or more missense variant (207 individuals carried both variant types), with the five most common protein changes being R175H (5.3%), R273C (3.2%), R248Q (3.2%), R273H (3.2%) and R282W (2.7%) (Supplementary Table 1). *PIK3CA* was the second most frequently altered gene, with mutations found in 19.8% of patients (2,750 of 13,880), occurring most frequently in uterine corpus endometrial carcinoma (53.5%), ovarian endometrioid adenocarcinoma (49.0%), breast invasive carcinoma (42.2%), uterine corpus endometrial serous carcinoma (38.1%) and colon adenocarcinoma (26.5%). The most commonly mutated codons in *PIK3CA* were E545 and H1047. Over 69.9% of all mutations in this gene were found in the five well-characterized hotspots^2^. While currently indicated for testing in breast invasive carcinoma only, *PIK3CA* mutations were present across multiple tumor types, suggesting that clinical trials with *PIK3CA* inhibitors could be considered in the future, if clinically appropriate. Other genes such as *APC*, *KRAS*, *VHL* and *IDH1* were highly enriched for mutations in only one or two tumor types. Our pancancer analysis is concordant with other large-scale sequencing endeavors^10^ such as ICGC and TCGA, albeit with variations due to cancer type proportions, reflected by a higher proportion of colon and rectum adenocarcinoma, and sarcoma in our cohort (Fig. 2a). The sequencing of a large number of ovarian tumor samples (*n* = 498) allowed further subtype classification, with a high prevalence of *TP53* variants being identified in high-grade serous carcinoma (89.8% of cases), *PIK3CA* variants in ovarian endometrioid adenocarcinoma (49.0%) and *KRAS* variants in low-grade ovarian serous carcinoma (33.3%).

### Fusions and CNAs

A high prevalence of amplifications or losses was found in *TP53, CDKN2A, MYC, CDKN2B* and *PTEN* across all cancer types (Fig. 4). Glioblastoma multiforme, low-grade glioma, head and neck squamous cell carcinoma, mesothelioma and sarcoma (Fig. 4 and Extended Data Fig. 1b) demonstrated the highest number of clinically relevant CNAs. With increased targeted therapies, molecular tests for different mutation types, including fusions, have become standard of care^15^. For instance, *NTRK* fusions (across all cancer types) but also other kinase fusions (for example, *ALK*, *ROS* and *RET* for lung cancers), are now included in the NGTDC. Although only a small percentage of patients test positive for specific fusion, the presence of a mutation can be critical for disease classification. A prime example is found in mesenchymal chondrosarcomas, where *HEY1*–*NCOA2* fusions are exclusive to that subtype. Indeed, sarcomas had the highest prevalence of tumors (13%) with clinically relevant SV findings^16^ (Fig. 4 and Extended Data Fig. 1c).

### Germline findings

Unlike targeted panel tests that are frequently performed on tumor-only samples, paired tumor and normal WGS allows somatic and germline variants to be detected together. The certainty of origin for a variant can have implications on patient management, such as family genetic testing or eligibility for treatment. Patients with ovarian high-grade serous carcinoma had the highest prevalence of actionable germline findings for SNVs and indels, with 13% of patients harboring variants in the *BRCA1* and *BRCA2* genes (Fig. 4 and Extended Data Fig. 1d; predicted truncating small variants or missense mutations with pathogenic classification in Clinvar are reported; for details, see the online Methods). Median age at tumor diagnosis is shown in Fig. 2c; as expected, there was a younger median age at tumor diagnosis in those patients with predisposing germline findings (Extended Data Table 1). Notably, patients with germline variants in mismatch repair (MMR) genes showed significantly earlier age at onset of colon adenocarcinomas, while patients with germline variants in homologous recombination repair genes showed significantly earlier onset in ovarian high-grade serous carcinomas and breast invasive carcinomas. This was also observed in kidney renal clear cell carcinoma with germline variants predominantly in the *VHL* gene. *DPYD* variants, linked to fluoropyrimidine toxicity, were present in 5–10% of participants, guiding the recommendations for dose omission or adjustment in the treatment of breast invasive carcinomas, colon, rectum, pancreatic adenocarcinomas and head and neck squamous cell carcinomas as recommended in the NGTDC.

### Pangenomic markers and mutational signatures

**Fig. 5:**
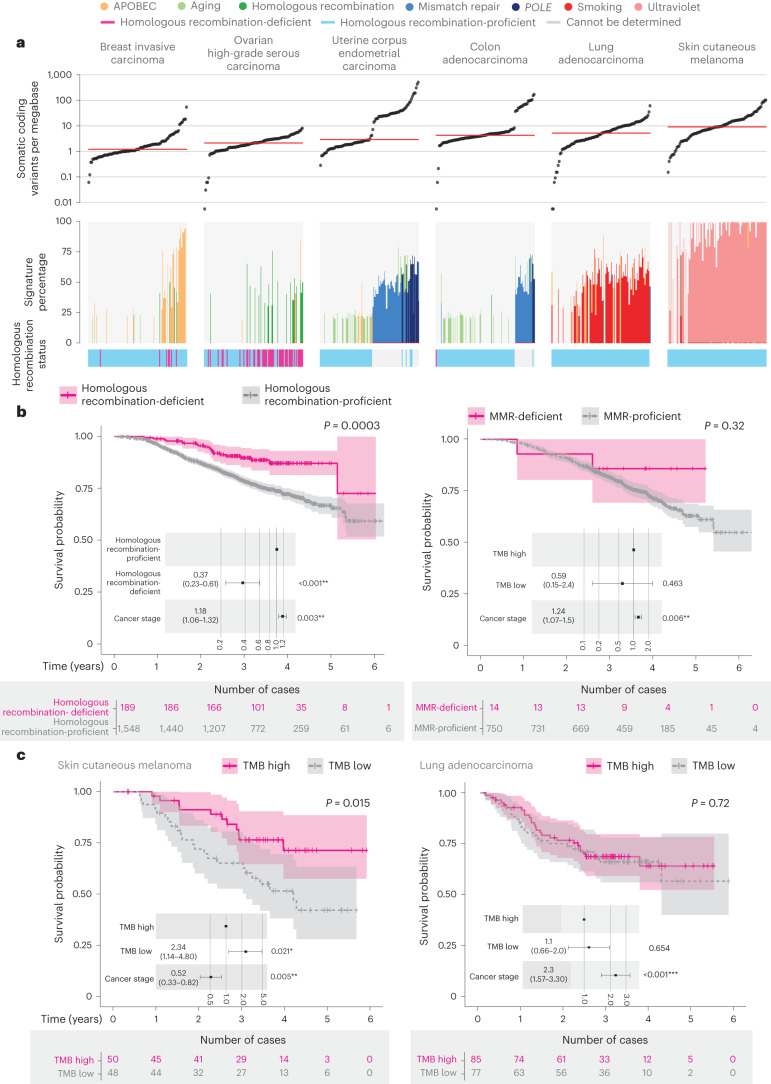
Predictive value of pangenomic markers derived from WGS data. **a**, Distribution of TMB and mutational signatures across six tumor types. (Samples that underwent PCR amplification during library preparation were excluded and the dataset for each tumor type was downsampled to 100 samples.) The horizontal red bar indicates the median TMB for each cancer type. Etiology definitions based on COSMIC (v.3) single-base substitution signatures: APOBEC activity, signatures 2 and 13; aging, signature 1; HRD, signature 3; MMR deficiency, signatures 6, 15, 20, 21, 26 and 44; *POLE* mutations, signatures 10a, 10b and 14; smoking, signatures 4 and 92; ultraviolet exposure, signatures 7a–d. Only signatures with more than 20% contribution are shown. Homologous recombination status is indicated in the bars below the signature plots. **b**,**c**, Kaplan–Meier estimates of overall survival with *P* values calculated using a stratified log-rank test. The numbers of patients at risk at different time points are indicated below the survival curves. The points and error bars on the embedded forest plots indicate the hazard ratios (HRs) with 95% confidence intervals (CIs), correspondingly. HRs, CIs and *P* values were calculated from Cox proportional-hazards models corrected according to cancer stage. Patients were stratified according to HRD status in cancers treated with platinum chemotherapy (*n* = 1,737, left, **b**); according to MMR signatures in cancers treated with immunotherapies (*n* = 764, right, **b**); according to high and low TMB in skin cutaneous melanoma (*n* = 98, left, **c**); and according to lung adenocarcinoma (*n* = 162, right, **c**). Exact *P* values can be found in Supplementary Table 2.

TMB has been cited as a potential biomarker^17^ and in this dataset we observed significant variation across and within cancer types. In line with previous reports^18^, we found that skin cutaneous melanoma and lung adenocarcinoma had the highest average TMB (Fig. 5a). Colon adenocarcinoma and uterine corpus endometrial carcinoma showed variability in the presence or absence of microsatellite instability or hypermutation caused by *POLE* mutations (see alignment with corresponding mutational signatures).

**Extended Data Fig. 2:**
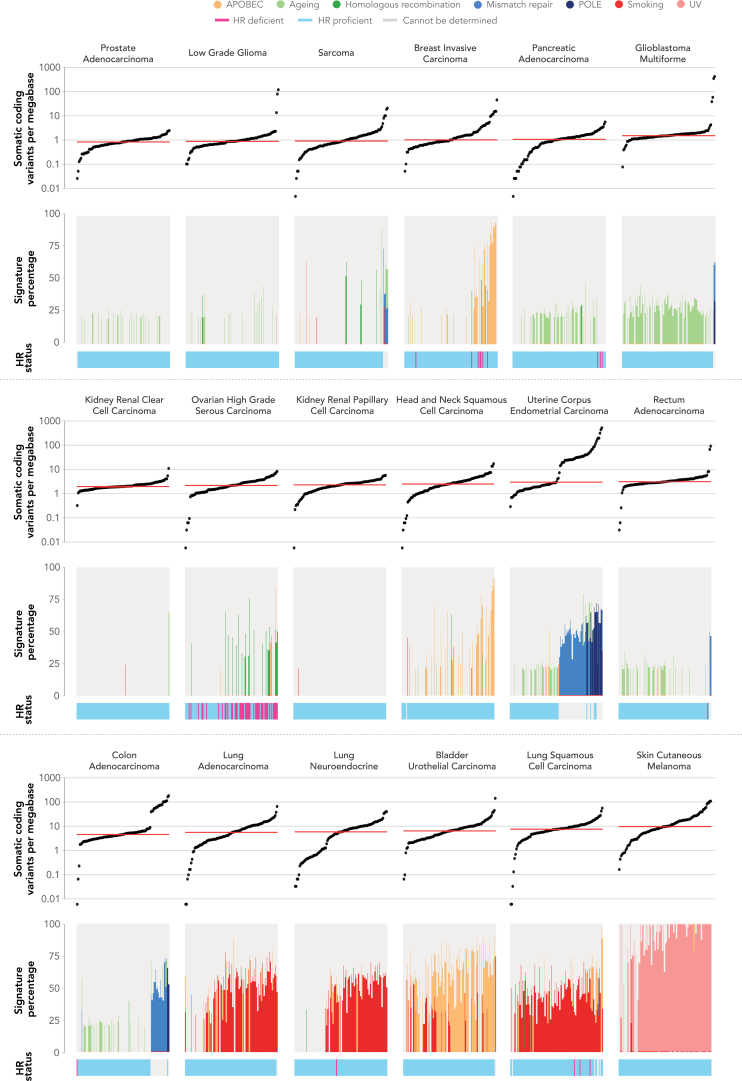
Distribution of tumor mutation burden (TMB) and mutational signatures across tumor types. Assignment of signatures to known etiologies matches Fig. 3.

When examining mutational signatures (COSMIC v.3) with well-described etiologies, we observed expected frequencies within certain cancer types^19^ (Fig. 5a and Extended Data Fig. 2). As expected, APOBEC signatures 2 and 13 were associated with breast invasive carcinoma, head and neck squamous cell carcinoma, bladder urothelial carcinoma and lung adenocarcinoma; smoking signatures 4 and 92 with lung cancers (lung adenocarcinoma, lung neuroendocrine and lung squamous cell carcinoma); and ultraviolet signature (signatures 7a–d) with skin cutaneous melanoma. DNA MMR signatures 6, 15, 20, 21, 26 and 44 were enriched in microsatellite instability-high colon adenocarcinoma and uterine corpus endometrial carcinoma (Fig. 5a).

HRD status was defined by two genome-wide mutational scar-based pancancer classifiers, CHORD^20^ and HRDetect^21^. The two algorithms demonstrated 99.2% concordance in our sample cohort (Methods). Ovarian high-grade serous carcinoma showed the highest prevalence of HRD (40%). While PARP inhibitors are currently only indicated for use in ovarian tumors with HRD, HRD was also detected at low prevalence in other cancers that could potentially access PARP inhibitors via clinical trials or compassionate access pathways.

### Clinical utility of WGS

Overall, these findings demonstrate the ability of WGS data to fully characterize the clinical genomic landscape of a tumor. A single test can report somatic SNVs, gene fusions and CNAs, along with potentially pathogenic germline mutations, and pangenomic markers such as mutational signatures and TMB (Fig. 4). In the Supplementary Information, we provide examples of WGA results as provided to NHS GMC Laboratories. For example, in a patient with ovarian high-grade serous carcinoma, a somatic *TP53* SNV was identified, consistent with the diagnosis, along with a germline *BRCA1* variant and somatic *BRCA1* copy number (CN) loss driving HRD, which was subsequently supported by the HRD analysis. Similarly, in another case, in a patient with endometrial cancer, MMR deficiency signatures were identified in combination with high TMB, along with a *PMS2* pathogenic germline variant, a somatic *PMS2* start–loss mutation and a pharmacogenomic (germline) variant in the *DPYD* gene (associated with toxicity to fluoropyrimidines). These examples demonstrate specific instances where the identification of different types of mutations and pangenomic markers were clinically relevant.

### Pangenomic markers and outcomes from real-world data

Through the link of the WGS data with longitudinal life course clinical data (SACT and ONS), we assessed treatment outcomes for patients stratified according to pangenomic markers (Fig. 5b and Supplementary Table 2). As shown in Fig. 5b, in patients treated with platinum therapies, HRD predicted better outcome (*n* = 189, *P* < 0.001, HR = 0.37, CI = 0.23–0.61), primarily in patients with invasive breast carcinomas (*n* = 44, 23.3%) and ovarian high-grade serous carcinomas (*n* = 126, 66.7%). Immunotherapy outcomes in MMR-deficient cases (*n* = 14) were inconclusive because of small numbers. We then evaluated TMB as a prognostic marker^22^ and a significant difference in survival (*P* = 0.015, HR = 2.34, CI = 1.14–4.80) was observed for those patients with TMB in the lowest quartile (median of 3.8 nonsynonymous small variants per Mb) compared with the highest quartile (median of 20.98 nonsynonymous small variants per Mb) in those diagnosed with skin cutaneous melanoma (Fig. 5c and Supplementary Table 2). Interestingly, a significant difference was not observed in lung adenocarcinoma (*P* = 0.72), where the lowest and highest quartile median TMB values were 2.2 and 10.5 nonsynonymous small variants per Mb, respectively. This may indicate that the level of TMB is relevant in prognosis and supports the need for further refining of pangenomic biomarkers as both prognostic and predictive for immunotherapy response, as highlighted in previous studies^23,24^.

### Co-occurrence of small variants and CNAs

**Fig. 6:**
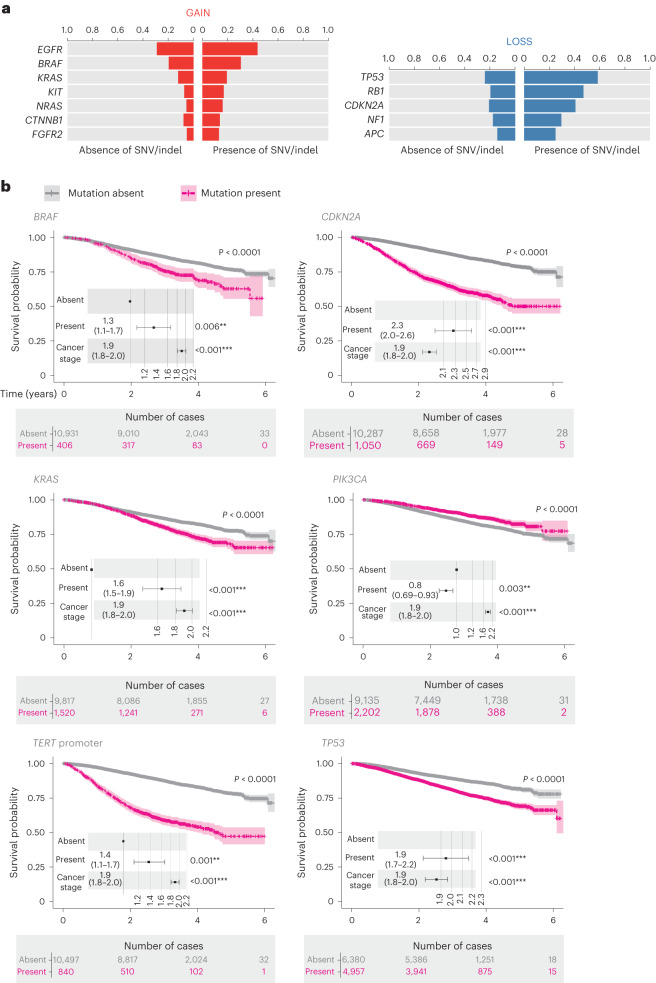
Prognostic value of small variants and CNAs from WGS data. **a**, Co-occurrence of CNAs and small variants in clinically actionable genes. The bars represent the proportion of cases with CNA in the subset of cases with or without small variants (SNV or small indels) in clinically actionable genes. Oncogenes and tumor suppressor genes were tested for gain (red) or loss (blue) of at least one copy of the corresponding gene, respectively. **b**, Kaplan–Meier estimates of overall survival with *P* values calculated using a stratified log-rank test. The numbers of patients at risk at different time points are indicated below the survival curves. Points and error bars on the embedded forest plots indicate HRs with 95% CIs, correspondingly. HRs, CIs and *P* values were calculated from Cox proportional-hazards models corrected according to cancer stage. Patients were stratified according to the mutational status of genes indicated for testing in NGTDC across all cancer types (*n* = 11,337). Exact *P* values can be found in Supplementary Table 2.

The co-occurrence of SNVs, indels and CNAs is well documented^25^. With WGS, we were able to explore the co-occurrence of CNAs and somatic small variants impacting cancer genes in the NGTDC. We divided cases into those with and without small variants for each gene and then compared the frequency of CNAs for each gene across these two groups (Fig. 6a and Supplementary Table 3). After multiple-testing correction, we found that 12 genes displayed a significant difference in the frequency of copy alterations. We confirmed previous findings, namely, that *EGFR*^26^ and *KIT*^27^, in specific cancer types, tended to be amplified when a putative activating SNV was present. The role of copy gains on certain oncogenes has long been debated and our analysis found that there was a significant co-occurrence of gains in the presence of small variants affecting *BRAF*, *KRAS*, *NRAS*, *CTNNB1* and *FGFR2*. We also found that five tumor suppressor or dual-role genes had significantly higher frequencies of copy loss in the presence of somatic small variants, including established examples such as *TP53* (ref. ^28^), *RB1* (ref. ^29^), *CDKN2A*^30^ and *APC*^25^, further emphasizing the value of interpreting different types of variants concurrently.

### Survival analysis using real-world data

**Extended Data Fig. 3:**
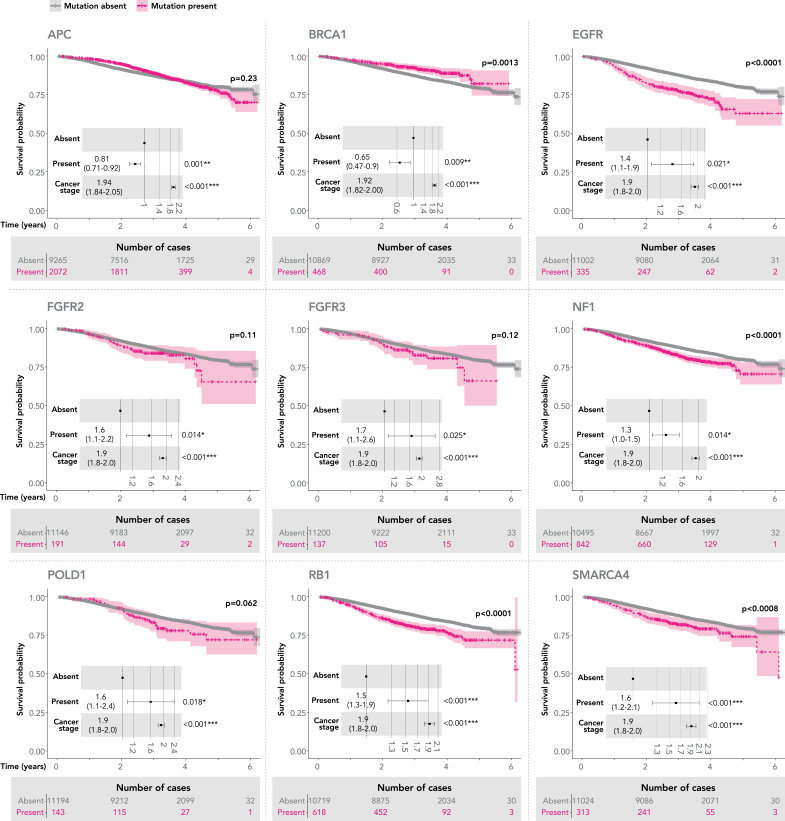
Kaplan-Meier estimates of overall survival with p-values calculated using a stratified log-rank test. Numbers of patients at risk at different time points are indicated below the survival curves. Points and error bars on the embedded forest plots indicate hazard ratios (HR) with 95% confidence intervals (CI), correspondingly. HR, CI and p-values are calculated from cox proportional hazards models corrected by cancer stage. Patients are stratified by mutational status of genes indicated for testing in NGTDC across all cancer types (n = 11337). Exact p-values can be found in Supplementary Table S2.

We next assessed overall survival in all 33 cancer subtypes stratified according to the presence or absence of mutations in 40 NGTDC-indicated genes (protein-altering small variants (SNVs and indels) as well as homozygous deletions in tumor suppressor genes were included). Clinical data from secondary data sources such as HES and ONS provided survival data. Kaplan–Meier and Cox proportional-hazards analyses were performed on our pancancer cohort. After correcting for stage and multiple testing, 15 genes affected overall survival (Fig. 6b and Extended Data Fig. 3). The gene that affected patient outcome most severely was *CDKN2A* (*P* < 1 × 10^−10^, HR = 2.3, CI = 2.0–2.6), which corresponds to its association with high-grade disease and poor prognosis in some cancer subtypes, such as glioma^31^ and soft-tissue sarcoma^32^. Our results agree with previously reported prognostic associations for specific tumor types, for example, poor prognosis for *KRAS* mutants in colorectal cancer^33^ and non-small cell lung cancer^34^ or *TP53* mutations in non-small cell lung cancer^35^. Mutations in *PIK3CA* were associated with favorable outcomes, in keeping with reports in the literature^36^.

## Discussion

The 100,000 Genomes Project established the infrastructure and resources for linking genomic and longitudinal clinical life course data. Our findings from the Cancer Programme aided the selection of genomic targets in the NHS National Genomic Test Directory. Evaluation of WGS data provided support for the commissioning of clinical WGS for sarcoma, glioblastoma, ovarian high-grade serous carcinoma and triple-negative breast cancers, to detect different types of mutations, including pangenomic markers, with a single test to inform clinical care. The infrastructure generated from the 100,000 Genomes Project has been incorporated into the NHS GMS to enable standardized molecular characterization of tumors and to extend the clinical benefit of prospective molecular characterization to more patients with cancer. Consistent with previous studies^37^ we report a high prevalence of genetic variants used to stratify patients toward approved therapies and clinical trials across different cancer types. Our approach aligns with similar programs in other countries, such as St. Jude Children’s Research Hospital^38^ in the USA, BC Cancer in Canada^39^, Zero Childhood Cancer Program in Australia^40^, France Médecine Génomique^41^ and Genomic Medicine Sweden^42^. These initiatives are either ongoing and have yet to publish on their cohort or represent a smaller cohort of childhood cancers.

Our study only included WGS data and while genomics may provide a valuable starting point for molecular stratification of cancer, it is likely that other modalities, such as cell-free DNA, RNA sequencing, methylation and gene expression profiling, proteomics, long-read sequencing and single-cell sequencing will mature toward clinical use. As such, we envisage the inclusion of multi-omics data alongside longitudinal life course data and the integration of multimodal molecular and clinical data, including digital pathology and radiology, to maximize the benefit of precision cancer care for patients^43,44^.

As genomic testing becomes more widespread, it is essential to combine these data with real-world clinical and treatment data. This integration is crucial to advancing our understanding of the long-term impact of clinical cancer genomics on patient outcomes. In this study, we demonstrated the value of linked real-world data in evaluating outcomes and mirroring adverse molecular markers from clinical trials. The accumulation of genomic data alongside electronic health data included in cancer registries, such as staging, pathology and treatment, and outcomes, enriches the dataset and may further refine the selection of biomarkers. The co-occurrence of variants in the same gene, or the coexistence of mutations in different genes, are likely to enhance the prognostic and predictive value of biomarker selection and may detect longer-term latent signals of benefit or harm and aid clinical and regulatory decision-making^43^. The therapeutic implications associated with the co-occurrence of CNAs and somatic small variants are unclear, and this level of genomic information may not readily be available from large cancer panel data^45^. We present a broad survival analysis at the gene level; as the dataset expands, it will be possible to examine these data further to establish prognostic and predictive implications for specific variants, as observed with *KRAS* variants^46,47^.

Yet, challenges remain in implementing clinical WGS in the NHS in England not least because of the overall cost compared to large gene panel testing. Providing a cutting-edge UK genomics service requires not only the sequencing and analytical infrastructure, but the consideration of operational requirements (such as improvements in tissue pathways and turnaround times to inform clinical decision-making) together with local pathway transformation and the development of knowledge and skills of the multiprofessional workforce supporting cancer care.

WGS results are discussed at multidisciplinary Molecular Tumor Boards or GTABs to evaluate somatic and germline variants, determine clinical actionability and provide clinical recommendations. GTABs have a vital role in ensuring that actionable results are communicated to treating teams and clinicians, while also exploring eligibility for approved therapies and clinical trials^48^. A well-designed, well-structured GTAB has a key role in the clinical interpretation of cancer genomic testing, guiding clinicians in decision-making through recommendations, facilitating clinical trial enrollment and potentially enhancing outcomes^49,50^. This approach aligns with adaptive basket trials such as DETERMINE^51^, which has been established to evaluate licensed treatments in unlicensed indications similar to the DRUP trial^12^. The aim is to enable more equitable and comprehensive molecular testing within the NHS and to optimize cancer care by identifying all clinically relevant mutations for a specific cancer (as shown in Fig. 4) and their relationship to approved precision medicines, but also to ensure that patients are fully considered for clinical research and trials because of this genomic testing and to explore clinical trial options, including the use of repurposed well-known and well-characterized drugs.

The Research Environment, a platform built by Genomics England and NHSE, allows approved researchers secure access to genomic data and associated health data. It has allowed advances in fundamental research, such as the discovery of cancer driver genes^52^, mutational signatures^53^ or changes in clinical practice driven by availability of WGS testing^54,55^.

Our findings underscore the potential for these data to provide additional prognostic insights based on the absence or presence of specific mutations. As data accumulate within the Research Environment with linkage of genomic, clinical and outcome data, more refined analyses using real-world data can take place, aided by more comprehensive tumor profiling. This will enable further refinement of prognostic and predictive molecular markers, not only with combinations of different genomic alterations, but beyond genomics, including emerging technologies to expand the reach of precision oncology to improve cancer outcomes.

## Methods

### Sample collection

The sample collection and DNA extraction requirements are described in the Sample Handling Guidance (v.4.0) available at https://files.genomicsengland.co.uk/forms/Sample-Handling-Guidance-v4.0.pdf. A total of 10 μg germline DNA and at least 1.3 μg tumor DNA were required for Illumina TruSeq PCR-free library preparation to be performed. PCR-based library preparation was used when insufficient DNA could be obtained for PCR-free sequencing, with a minimum requirement of 500 ng. Optimized formalin-fixed tumor tissue was allowed for WGS under exceptional circumstances, where tumor size limited availability of fresh tissue, or if no tumor was present in the fresh-frozen sample.

### Analytical bioinformatics pipeline

For full details of the bioinformatics pipeline, see the Cancer Genome Analysis Technical Information Document at https://files.genomicsengland.co.uk/forms/Cancer-Analysis-Technical-Information-Document-v1-11-main.pdf.

#### Quality of sequencing data

All samples were sequenced on the HiSeq platform to an average coverage of 100× for tumor and 30× for normal. The following checks were implemented to ensure sample quality: normal samples had more than 85 Gb and tumor samples had more than 210 Gb of high-quality sequencing data (base quality greater than 30, duplicated reads removed); normal samples had more than 95% of the autosomal genome covered at 15× or more after removing reads with mapping quality lower than 10; normal samples had cross-patient contamination lower than 3% as assessed using VerifyBamID; tumor samples had cross-patient contamination lower than 2.5% and normal tumor sample pair originating from the same patient as assessed using ConPair; the quality of the sequencing data was monitored using principal component analysis based on the following metrics: percentage of reads mapped to the reference genome, proportion of chimeric DNA fragments, median fragment size, unevenness of local genome coverage and percentage of reads missing from AT-rich or GC-rich genomic regions (AT and GC drop).

#### Mapping and variant calling

The Illumina North Star pipeline (v.2.6.53.23) was used for the primary WGS analysis. Read alignment against the human reference genome GRCh38 + decoy + Epstein–Barr virus was performed with ISAAC (v.iSAAC-03.16.02.19). We acknowledge deficiencies in the ISAAC alignment software for precise variant allele frequency estimates^56^ and for tumor evolution analysis and note that all genomes from the 100,000 Genomes Project were recently realigned with the Illumina Dragen platform (data available in the Research Environment). Germline small variant calling was performed using Starling (v.2.4.7) and somatic small variant calling was performed using Strelka (v.2.4.7). In addition to default Strelka filters, the following additional filters were applied to reduce the false positive rate in the set of somatic variants used as an input into the calculation of TMB and mutational signatures: (1) variants with a population germline allele frequency above 1% in the Genomics England or gnomAD datasets; (2) recurrent somatic variants with a frequency above 5% in the Genomics England dataset; (3) variants overlapping simple repeats as defined by Tandem Repeats Finder; (4) small indels in regions with high levels of sequencing noise where at least 10% of the base calls in a window extending 50 bases to either side of the indel call were filtered out by Strelka because of poor quality; (5) SNVs resulting from systematic mapping and calling artifacts with a Fisher’s exact test Phred score lower than 50. The flagging of systematic mapping and calling was performed by testing whether the ratio of tumor allele depths at each somatic SNV site were significantly different to the ratio of allele depths at this site in a panel of normals. The panel of normals consisted of a cohort of 7,000 non-tumor genomes from the Genomics England dataset; at each genomic site only individuals not carrying the relevant alternate allele were included in the count of allele depths. Variants flagged with any of the above internal filters were not removed from the WGA results of clinically actionable variants but were labeled in the output shared with clinical scientists.

CNAs were identified with Canvas v.1.3.1. Manta (v.0.28.0) was used to call SVs and long indels (more than 50 bp), combining paired and split-read evidence for SV discovery and scoring.

Estimates of the accuracy of somatic variant calling in the 100,000 Genomes Project pipeline were produced as a requirement for accreditation under International Organization for Standardization no. 15189. We have provided ‘Bioinformatics Pipeline Validation. Cancer Report, September 2018’ as Supplementary Information and have summarized the findings in Supplementary Table 4. Extensive validation and functional improvements of the pipeline for the NHS GMS will be presented in a separate publication.

#### Annotation and reporting actionability

SNVs and small indels were left-aligned, trimmed, and multi-allelic variants decomposed, before annotation with Cellbase, using the Ensembl (v.90/GRCh38), COSMIC (v.v86/GRCh38) and ClinVar (October 2018 release) databases. Annotation of consequence types was carried out by a high-performance variant annotator within Cellbase; only variants annotated with a curated set of consequence types (stop gained or lost, start lost, frameshift variant, inframe insertion or deletion, missense variant, splice acceptor or donor variant, splice region variant) in canonical transcripts were reported.

Interpretation of CNAs took into account gene mode of action as defined in the COSMIC Cancer Gene Census (that is, oncogene or tumor suppressor gene). Where a gene had an ambiguous or unknown role in cancer, it was included in both oncogene and tumor suppressor categories. Gains in oncogenes were reported if CN was at least twice higher than the overall ploidy as defined by Canvas. The following scenarios were reported as losses in tumor suppressor genes: (1) homozygous deletions called by Canvas (CN = 0); (2) loss of heterozygosity (LOH) called by Canvas (CN = 1) or copy-neutral LOH, in combination with a nonsynonymous somatic small variant; and (3) Manta SVs with the potential to disrupt the gene coding region in combination with a nonsynonymous somatic small variant. Only samples with tumor purity greater than 30% were included in the CNA actionability analysis. For the co-occurrence of somatic small variants and CNAs analysis in Fig. 6a, gain of at least one copy for oncogenes or loss of at least one copy for tumor suppressor genes was counted as a CNA event.

Manta calls (break end, deletion, duplication or inversion) were further assessed for the potential to generate productive fusions using an in-house approach based on transcript orientation and consistency of reading frame across the SV breakpoint. SVs that were identified as out of frame or untranscribed were discarded. Potential inframe fusions and ambiguous events with a breakpoint in the coding exon or in the 5-′UTR of downstream partners were reported.

Germline variants listed in ClinVar as pathogenic or probably pathogenic with a rating of at least two stars and predicted protein-truncating variants in genes for which the mechanism of pathogenicity was loss of function (stop gained or lost, start lost, frameshift variant, splice acceptor or donor variant) were reported for a subset of cancer predisposition genes indicated for germline testing in NGTD.

Within the context of the 100,000 Genomes Project Cancer Programme, all variants returned to GMCs were reviewed within GTABs to classify further if variants were pathogenic or probably pathogenic (germline) or oncogenic or probably oncogenic (somatic) and to provide clinical recommendations where appropriate.

#### Signatures and TMB

For each tumor sample, frequencies across all SNV trinucleotide contexts were calculated using VCF files that were filtered for potential false positive variants (see the variant calling section) and the contribution of each of the COSMIC (v.3) single-base substitution signatures to the overall mutational burden observed in the tumor was derived using decomposition by the SigProfiler suite of tools^57^. Etiology definitions were based on the following signature combinations: APOBEC activity, signatures 2 and 13; aging, signature 1; MMR deficiency, signatures 6, 15, 20, 21, 26 and 44; *POLE* mutations, signatures 10a, 10b and 14; smoking, signatures 4 and 92; ultraviolet exposure, signatures 7a–d. Signature 14 (reported with the etiology ‘concurrent polymerase epsilon mutation and defective DNA MMR’) was not included in the MMR deficiency group to avoid double counting in the MMR and *POLE* groups. Including SBS14 in the MMR group would change MMR status for 9 of 13,880 tumors and would only increase the number of MMR^+^ tumors in our cohort by 0.81%. For a given etiology, if the final combined signatures summed to less than 20%, the signature was assigned to ‘other’. Tumors were classified with MMR deficiency if the total contribution of MMR signatures was more than 20%. HRDetect^21^ is a logistic regression classifier that computes a probability score of HRD based on microhomology deletions, SNV and SV mutational signatures, and LOH score. HRD status using HRDetect was retrieved from a previous publication^53^. The CHORD algorithm is a random forest-based classifier that incorporates counts of different variant types as input (SNVs, microhomology deletions and SVs) and does not require an intermediate mutational signature extraction step)^20^. HRDetect and CHORD were trained on the ICGC and Hartwig Medical Foundation cohorts, respectively. The two algorithms returned concordant results for 99.2% of samples in our cohort (10,764 of 10,854) and CHORD results were used for the figures. TMB was calculated as the total number of nonsynonymous high-confidence somatic small variants per megabase of coding sequence (see the variant calling section for the filtering method used).

### Description of clinical data resources

A minimal set of patient and sample data was collected from GMCs at the time of DNA sample submission through OpenClinica v.3.4, for example, tumor type, year of birth, tissue source, self-reported gender. For the purposes of the analysis, self-reported gender was cross-validated with biological sex inferred using the ratio of mean sequencing coverage of sex chromosomes and mean sequencing coverage of autosomes. Assigned biological sex was used in the bioinformatics pipeline as an input for variant calling. Secondary clinical information was gathered from NHSE and Public Health England (PHE)/NCRAS. From NHSE, HES data were used to obtain details of all commissioned activity during admissions; mortality information was obtained from the ONS registry data for cancer registrations and deaths inside and outside of hospitals. From PHE/NCRAS, the av_tumor table was used to obtain tumor date of diagnosis, together with histology and morphology codes. The SACT table provided information on the date and types of treatment. All datasets were accessed via the National Genomics Research Library using LabKey.

### Linking genomic data with secondary data sources

Hematological tumors, pediatric tumors and carcinomas of unknown primary origin were considered to be outside the scope of the study and were removed before tumor selection. Secondary data from the PHE/NCRAS tumor catalog (av_tumor), and NHS Digital HES data were used to corroborate the clinical data submitted by the GMCs.

The av_tumor dataset was linked to genomic data on the basis of the participant identifier. Tumors labeled as either benign or in situ were removed from the selection process, leaving only malignant, unknown or NA (the latter being the case for Genomics England participants not present in the av_tumor dataset). Where av_tumor data were available for a participant, they were used to confirm the tumor type submitted by the GMC. For cases where the av_tumor data did not match the GMC submission, or data were not present, HES Admitted Patient Care data were used to select the closest relevant hospital appointment involving a primary diagnosis of cancer (based on International Statistical Classification of Diseases and Related Health Problems, 10th Revision (ICD-10) code) to the clinical sample time submitted by the GMC. If the ICD-10 code for that appointment was considered a match to the tumor type submitted by the GMC, the HES data were deemed as corroborating the GMC submission.

Where HES data did not corroborate the tumor type submitted by the GMC, three additional approaches were used: (1) for primary tumors, a curated set of HES operation codes was used to match the tumor type submitted by the GMC and the HES data if the operation date exactly matched the sampling date of the tumor submitted to Genomics England; (2) for non-primary tumors that were identified as colorectal by the av_tumor data, and as either hepato-pancreatobiliary, endometrial carcinoma or lung in the GMC submission, more flexible HES ICD-10 matching was allowed provided the date difference between the HES appointment date and tumor sampling date submitted by the GMC was fewer than 7 days; (3) for a small number of remaining samples, ICD-10 and morphology data submitted by the GMC were used to corroborate tumor type.

Tumor stage was obtained from the NCRAS dataset. Where stage_best was present in av_tumor and the date in the diagnosis database column was fewer than 365 days from the clinical sample time submitted by the GMC, stage_best was used (simplified to stages 1, 2, 3 and 4) (11,618 of 13,880, 83.7%). Tumors submitted as metastatic were assigned stage 4 by default. FIGO (Fédération Internationale de Gynécologie et d’Obstétrique) stage was used for ovarian- and endometrium-related clinical indications and Dukes’ staging was used for colon and rectum adenocarcinomas (both obtained from the av_tumor table). In total, stage information was obtained for 12,040 of 13,880 (86.7%) tumors.

### Survival analysis

All survival analyses were performed in R using the survminer and survival libraries. Specifically, the survfit and ggsurvplot functions were used to create the Kaplan–Meier plots, and coxph for the Cox proportional-hazards models. The ggforest function was used to create the forest plots. Date of death was obtained from the ONS data. Where a death was not recorded for an individual, treatment and operation event dates were obtained from the HES dataset and used to determine the last date an individual was seen to enable right-censoring of the data.

### Ethics

The research described in this manuscript complies with all relevant ethical regulations. Approval for the project was obtained from the East of England-Cambridge South Research Ethics Committee (Research Ethics Committee reference 14/EE/1112, Integrated Research Application System ID: 166046)^58,59^. Participants were selected on the basis of having been identified by healthcare professionals and researchers within the NHS as having a cancer diagnosis. Participants were recruited across 13 NHS GMCs and written informed consent was obtained from participants.

### Reporting summary

Further information on research design is available in the Nature Portfolio Reporting Summary linked to this article.

## Online content

Any methods, additional references, Nature Portfolio reporting summaries, source data, extended data, supplementary information, acknowledgements, peer review information; details of author contributions and competing interests; and statements of data and code availability are available at 10.1038/s41591-023-02682-0.

### Supplementary information

Supplementary Information Anonymized whole-genome analysis results for two participants. Bioinformatics pipeline validation. Cancer report, September 2018.

Reporting Summary

Supplementary Tables 1–20 This workbook contains 20 tables: Tables 1–4 supplement the main text; Tables 5–20 contain the processed aggregated data used to generate the figures.

## Figures and Tables

**Figure:**
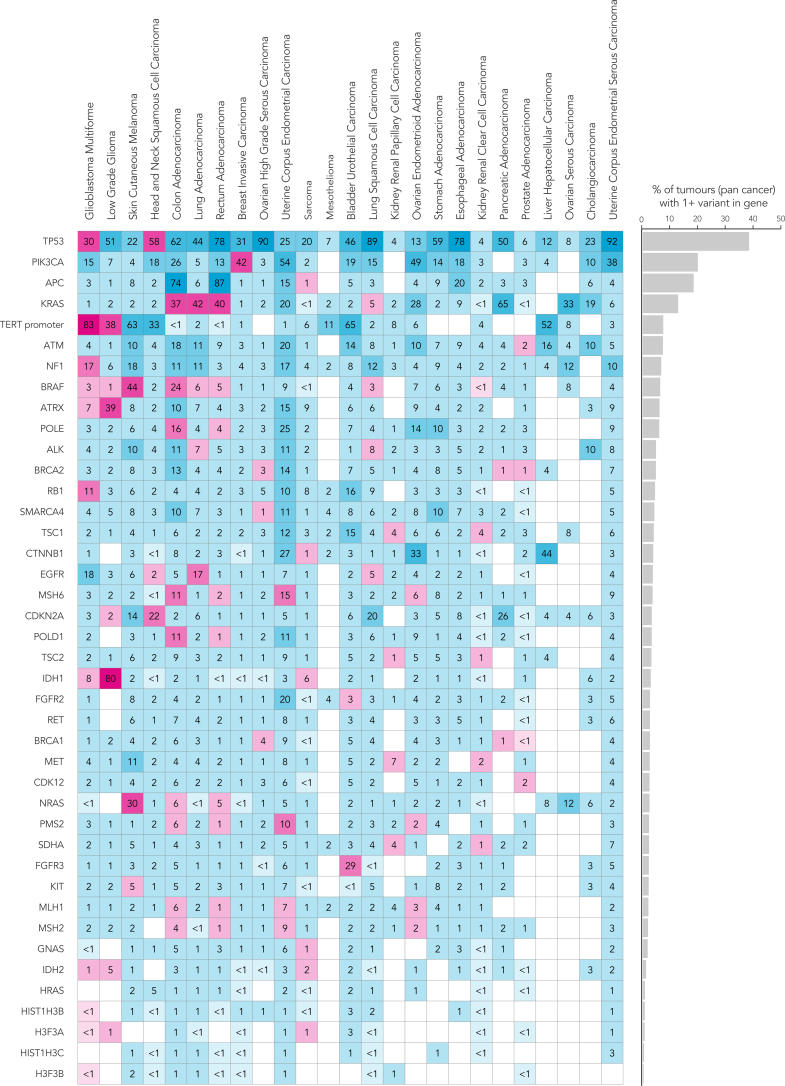
Extended Data Fig. 1A (SNV/Indel)

**Figure:**
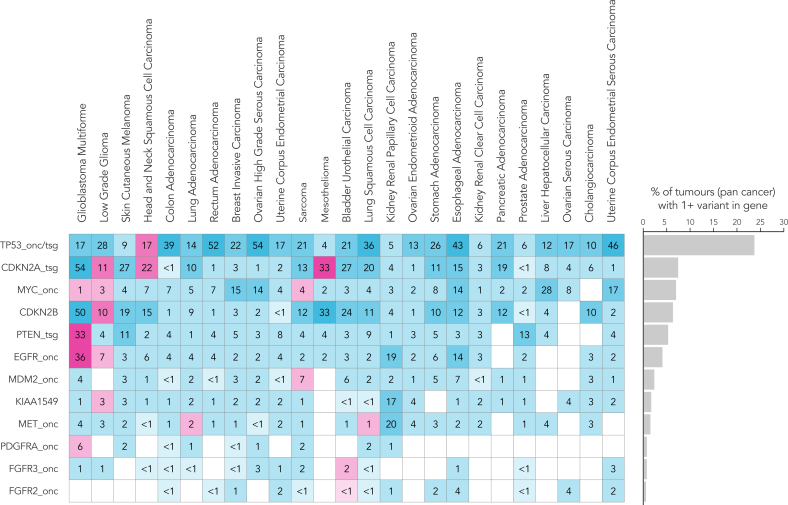
Extended Data Fig. 1B (CNA)

**Figure:**
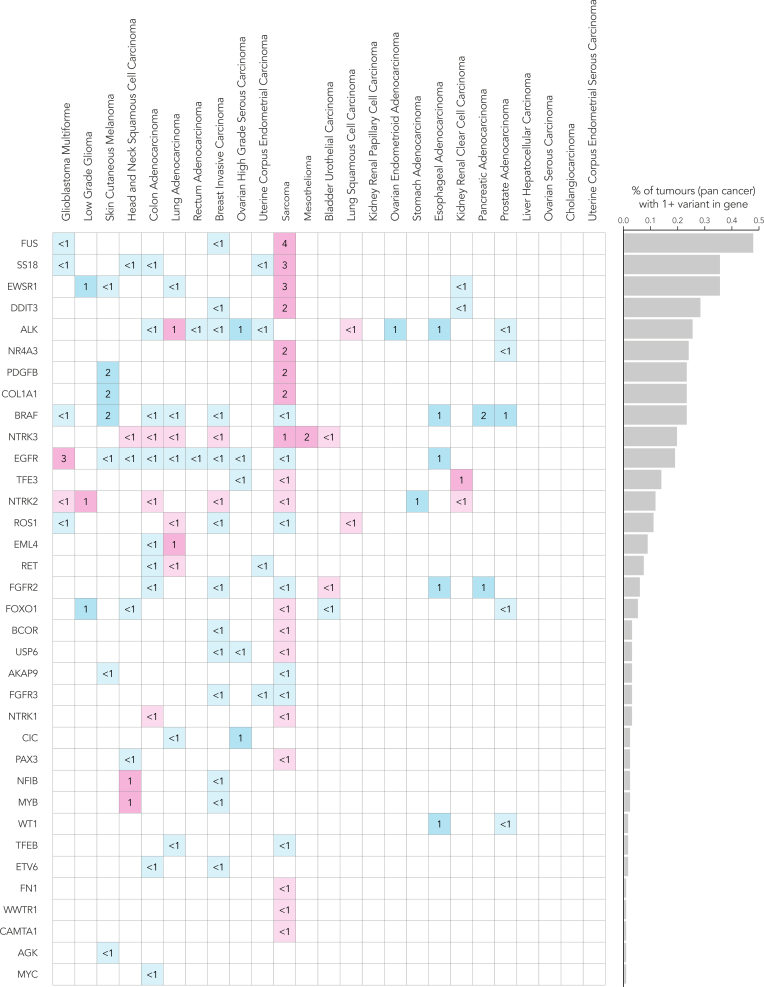
Extended Data Fig. 1C (SV)

**Figure:**
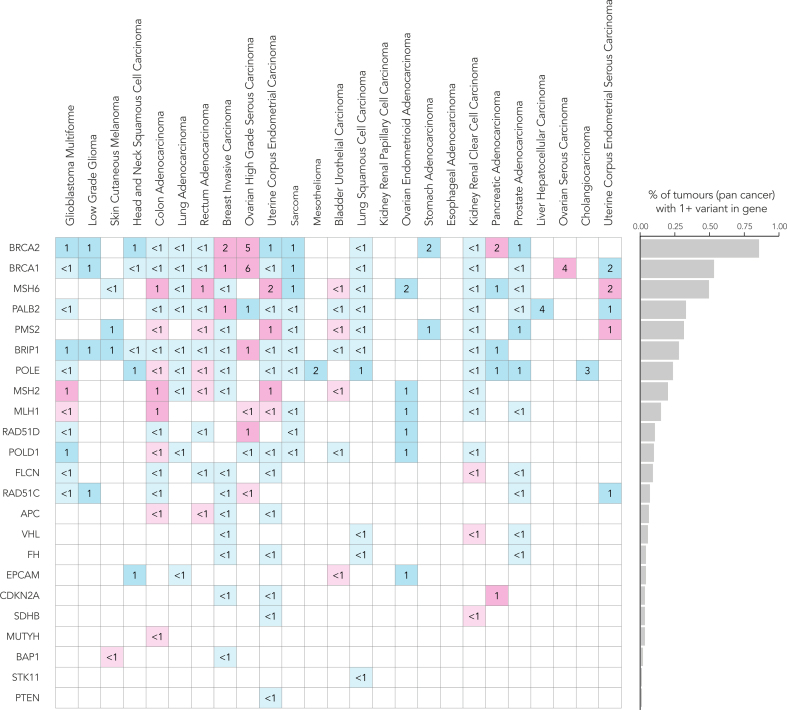
Extended Data Fig. 1D (Cancer-predisposing germline)

**Extended Data Table 1:**
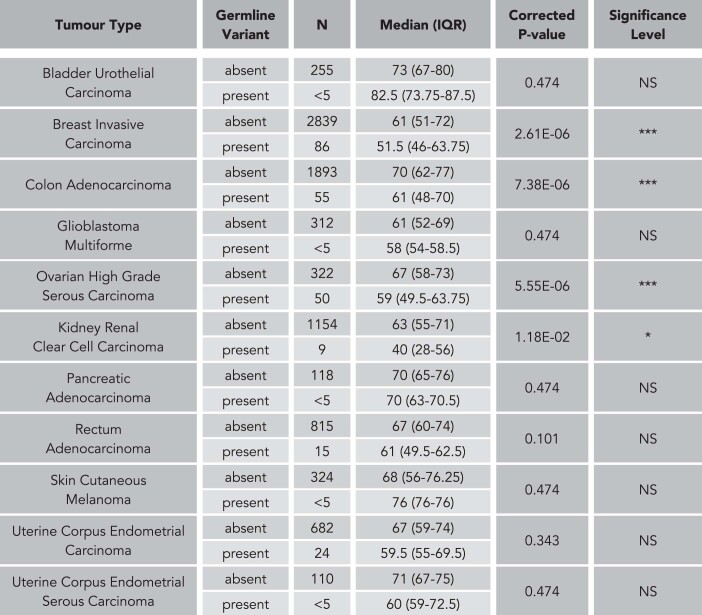
Median age and interquartile range (IQR) at diagnosis in the absence and presence of pertinent germline findings

Statistical significance of pertinent germline finding for early tumor onset was calculated by Wilcoxon rank-sum test with Benjamini–Hochberg multiple testing. ***P < 0.0001, *P < 0.05. Tumor types without germline variant testing indicated in the NGTDC were excluded.

## Data Availability

The data supporting the findings of this study are available within the Research Environment, a secure cloud workspace. Details on how to access data for this publication can be found at https://re-docs.genomicsengland.co.uk/pan_cancer_pub/. Additional processed aggregated data used to generate figures can be found in Supplementary Tables 5–20. To access the genomic and clinical data within this Research Environment, researchers must first apply to become a member of either the Genomics England Research Network (previously known as the Genomics England Clinical Interpretation Partnership, GECIP) (www.genomicsengland.co.uk/research/academic) or a Discovery Forum industry partner (www.genomicsengland.co.uk/research/research-environment). The process for joining the Genomics England Research Network is described at www.genomicsengland.co.uk/research/academic/join-gecip and consists of the following steps: (1) If it is not already participating, your institution will need to sign a participation agreement available at https://files.genomicsengland.co.uk/documents/Genomics-England-GeCIP-Participation-Agreement-v2.0.pdf and email the signed version to gecip-help@genomicsengland.co.uk; (2) once you have confirmed your institution is registered and have found a domain of interest, you can apply through the online form at www.genomicsengland.co.uk/research/academic/join-gecip. Once your Research Portal account is created you will be able to log in and track your application; (3) your application will be reviewed within ten working days; (4) your institution will validate your affiliation; and (5) you will complete our online Information Governance training and will be granted access to the Research Environment within 2 h of passing the online training. Data that have been made available to registered users include: alignments in BAM or CRAM format; annotated variant calls in VCF format; signature assignment; tumor mutational burden; sequencing quality metrics; summary of findings shared with the Genomic Lab Hubs; and secondary clinical data as described in this paper. Further details of the types of data available (for example, mortality, hospital episode statistics and treatment data) can be found at https://re-docs.genomicsengland.co.uk/data_overview/. Germline variants can be explored using the Interactive Variant Analysis Browser (https://re-docs.genomicsengland.co.uk/iva_variant/). The cohort of patients with cancer and longitudinal clinical information on treatment and mortality can be explored with Participant Explorer (https://re-docs.genomicsengland.co.uk/pxa/).

## Code availability

Details of the location of the code and data used to generate the figures can be found at https://re-docs.genomicsengland.co.uk/pan_cancer_pub/. The code is also available on GitLab (https://gitlab.com/genomicsengland/genomics_england_publications/100k_cancer_programme/) and has been uploaded to 10.5281/zenodo.8311292. Registered users will be able to copy and paste the code into RStudio in the Research Environment to recreate the figures. No bespoke mathematical algorithms were used in the analysis.

## References

[1] Cancer Incidence Statistics. *Cancer Research UK* www.cancerresearchuk.org/health-professional/cancer-statistics/incidence (undated).

[2] Zehir, A. et al. Mutational landscape of metastatic cancer revealed from prospective clinical sequencing of 10,000 patients. *Nat. Med.* **23**, 703–713 (2017). 28481359 10.1038/nm.4333 PMC5461196

[3] Smedley, D. et al. 100,000 Genomes Pilot on Rare Disease Diagnosis in Health Care—preliminary report. *N. Engl. J. Med.* **385**, 1868–1880 (2021). 34758253 10.1056/NEJMoa2035790 PMC7613219

[4] Turnbull, C. et al. The 100 000 Genomes Project: bringing whole genome sequencing to the NHS. *BMJ* **361**, k1687 (2018). 29691228 10.1136/bmj.k1687

[5] Turnbull, C. Introducing whole-genome sequencing into routine cancer care: the Genomics England 100 000 Genomes Project. *Ann. Oncol.* **29**, 784–787 (2018). 29462260 10.1093/annonc/mdy054

[6] Accelerating Genomic Medicine in the NHS. *NHS England* www.england.nhs.uk/long-read/accelerating-genomic-medicine-in-the-nhs (2022).

[7] National Genomic Test Directory. *NHS England* www.england.nhs.uk/publication/national-genomic-test-directories (2023).

[8] NHS England. *Board Paper* (2017); www.england.nhs.uk/wp-content/uploads/2017/03/board-paper-300317-item-6.pdf

[9] Berner, A. M., Morrissey, G. J. & Murugaesu, N. Clinical analysis of whole genome sequencing in cancer patients. *Curr. Genet. Med. Rep.* **7**, 136–143 (2019). 10.1007/s40142-019-00169-4

[10] Aaltonen, L. A. et al. Pan-cancer analysis of whole genomes. *Nature* **578**, 82–93 (2020). 32025007 10.1038/s41586-020-1969-6 PMC7025898

[11] Martínez-Jiménez, F. et al. Pan-cancer whole-genome comparison of primary and metastatic solid tumours. *Nature* **618**, 333–341 (2023). 37165194 10.1038/s41586-023-06054-z PMC10247378

[12] van der Velden, D. L. et al. The Drug Rediscovery protocol facilitates the expanded use of existing anticancer drugs. *Nature* **574**, 127–131 (2019). 31570881 10.1038/s41586-019-1600-x

[13] Cancer Incidence by Age. *Cancer Research UK* www.cancerresearchuk.org/health-professional/cancer-statistics/incidence/age (undated).

[14] Aran, D., Sirota, M. & Butte, A. J. Systematic pan-cancer analysis of tumour purity. *Nat. Commun.* **6**, 8971 (2015). 26634437 10.1038/ncomms9971 PMC4671203

[15] Zhong, L. et al. Small molecules in targeted cancer therapy: advances, challenges, and future perspectives. *Signal Transduct. Target Ther.* **6**, 201 (2021). 34054126 10.1038/s41392-021-00572-w PMC8165101

[16] Lanic, M.-D. et al. Detection of sarcoma fusions by a next-generation sequencing based-ligation-dependent multiplex RT–PCR assay. *Mod. Pathol.* **35**, 649–663 (2022). 35075283 10.1038/s41379-021-00980-x

[17] Chan, T. A. et al. Development of tumor mutation burden as an immunotherapy biomarker: utility for the oncology clinic. *Ann. Oncol.* **30**, 44–56 (2019). 30395155 10.1093/annonc/mdy495 PMC6336005

[18] Lawrence, M. S. et al. Discovery and saturation analysis of cancer genes across 21 tumour types. *Nature* **505**, 495–501 (2014). 24390350 10.1038/nature12912 PMC4048962

[19] Alexandrov, L. B. et al. Signatures of mutational processes in human cancer. *Nature* **500**, 415–421 (2013). 23945592 10.1038/nature12477 PMC3776390

[20] Nguyen, L., Martens, J. W. M., Van Hoeck, A. & Cuppen, E. Pan-cancer landscape of homologous recombination deficiency. *Nat. Commun.* **11**, 5584 (2020). 33149131 10.1038/s41467-020-19406-4 PMC7643118

[21] Davies, H. et al. HRDetect is a predictor of *BRCA1* and *BRCA2* deficiency based on mutational signatures. *Nat. Med.* **23**, 517–525 (2017). 28288110 10.1038/nm.4292 PMC5833945

[22] Xiao, D. et al. Analysis of ultra-deep targeted sequencing reveals mutation burden is associated with gender and clinical outcome in lung adenocarcinoma. *Oncotarget* **7**, 22857–22864 (2016). 27009843 10.18632/oncotarget.8213 PMC5008406

[23] Klempner, S. J. et al. Tumor mutational burden as a predictive biomarker for response to immune checkpoint inhibitors: a review of current evidence. *Oncologist* **25**, e147–e159 (2020). 31578273 10.1634/theoncologist.2019-0244 PMC6964127

[24] McGrail, D. J. et al. High tumor mutation burden fails to predict immune checkpoint blockade response across all cancer types. *Ann. Oncol.* **32**, 661–672 (2021). 33736924 10.1016/j.annonc.2021.02.006 PMC8053682

[25] Inoue, K. & Fry, E. A. Haploinsufficient tumor suppressor genes. *Adv. Med. Biol.* **118**, 83–122 (2017). 28680740 PMC5494974

[26] Sigismund, S., Avanzato, D. & Lanzetti, L. Emerging functions of the EGFR in cancer. *Mol. Oncol.* **12**, 3–20 (2018). 29124875 10.1002/1878-0261.12155 PMC5748484

[27] Cheng, L. et al. *KIT* gene mutation and amplification in dysgerminoma of the ovary. *Cancer* **117**, 2096–2103 (2011). 21523721 10.1002/cncr.25794

[28] Shetzer, Y. et al. The onset of p53 loss of heterozygosity is differentially induced in various stem cell types and may involve the loss of either allele. *Cell Death Differ.* **21**, 1419–1431 (2014). 24832469 10.1038/cdd.2014.57 PMC4131174

[29] Latil, A. et al. Loss of heterozygosity at chromosome arm 13q and *RB1* status in human prostate cancer. *Hum. Pathol.* **30**, 809–815 (1999). 10414500 10.1016/S0046-8177(99)90142-9

[30] Foulkes, W. D., Flanders, T. Y., Pollock, P. M. & Hayward, N. K. The *CDKN2A* (p16) gene and human cancer. *Mol. Med.* **3**, 5–20 (1997). 9132280 10.1007/BF03401664 PMC2230107

[31] Horbinski, C., Berger, T., Packer, R. J. & Wen, P. Y. Clinical implications of the 2021 edition of the WHO classification of central nervous system tumours. *Nat. Rev. Neurol.* **18**, 515–529 (2022). 35729337 10.1038/s41582-022-00679-w

[32] Bui, N. Q. et al. A clinico-genomic analysis of soft tissue sarcoma patients reveals *CDKN2A* deletion as a biomarker for poor prognosis. *Clin. Sarcoma Res.* **9**, 12 (2019). 31528332 10.1186/s13569-019-0122-5 PMC6739971

[33] Ozer, M. et al. Age-dependent prognostic value of *KRAS* mutation in metastatic colorectal cancer. *Future Oncol.* **17**, 4883–4893 (2021). 34758634 10.2217/fon-2021-0650 PMC8890131

[34] Aredo, J. V. et al. Impact of *KRAS* mutation subtype and concurrent pathogenic mutations on non-small cell lung cancer outcomes. *Lung Cancer* **133**, 144–150 (2019). 31200821 10.1016/j.lungcan.2019.05.015 PMC9348589

[35] Jiao, X.-D., Qin, B.-D., You, P., Cai, J. & Zang, Y.-S. The prognostic value of TP53 and its correlation with EGFR mutation in advanced non-small cell lung cancer, an analysis based on cBioPortal data base. *Lung Cancer* **123**, 70–75 (2018). 30089598 10.1016/j.lungcan.2018.07.003

[36] Kalinsky, K. et al. *PIK3CA* mutation associates with improved outcome in breast cancer. *Clin. Cancer Res.* **15**, 5049–5059 (2009). 19671852 10.1158/1078-0432.CCR-09-0632

[37] Priestley, P. et al. Pan-cancer whole-genome analyses of metastatic solid tumours. *Nature* **575**, 210–216 (2019). 31645765 10.1038/s41586-019-1689-y PMC6872491

[38] Ma, X. et al. Pan-cancer genome and transcriptome analyses of 1,699 paediatric leukaemias and solid tumours. *Nature* **555**, 371–376 (2018). 29489755 10.1038/nature25795 PMC5854542

[39] Pleasance, E. et al. Whole-genome and transcriptome analysis enhances precision cancer treatment options. *Ann. Oncol.* **33**, 939–949 (2022). 35691590 10.1016/j.annonc.2022.05.522

[40] Wong, M. et al. Whole genome, transcriptome and methylome profiling enhances actionable target discovery in high-risk pediatric cancer. *Nat. Med.* **26**, 1742–1753 (2020). 33020650 10.1038/s41591-020-1072-4

[41] Préindications d’Accès au Séquençage Génomique. *France Medecine Genomique 2025* https://pfmg2025.aviesan.fr/le-plan/indications-dacces-au-sequencage-genomique/ (undated).

[42] Sequencing of 7,000 Genomes in Swedish Clinical Practice in 2021 for Better Diagnosis and Treatment. *Genomic Medicine Sweden* https://genomicmedicine.se/en/2022/04/11/sequencing-of-7000-genomes-in-swedish-clinical-practice-2021-for-better-diagnosis-and-treatment (2022).

[43] Donoghue, M. T. A., Schram, A. M., Hyman, D. M. & Taylor, B. S. Discovery through clinical sequencing in oncology. *Nat. Cancer* **1**, 774–783 (2020). 35122052 10.1038/s43018-020-0100-0 PMC8985175

[44] Nogrady, B. How cancer genomics is transforming diagnosis and treatment. *Nature* **579**, S10–S11 (2020). 32214255 10.1038/d41586-020-00845-4

[45] Chandramohan, R. et al. A validation framework for somatic copy number detection in targeted sequencing panels. *J. Mol. Diagn.* **24**, 760–774 (2022). 35487348 10.1016/j.jmoldx.2022.03.011 PMC9302205

[46] Huang, L., Guo, Z., Wang, F. & Fu, L. *KRAS* mutation: from undruggable to druggable in cancer. *Signal Transduct. Target Ther.* **6**, 386 (2021). 34776511 10.1038/s41392-021-00780-4 PMC8591115

[47] van de Haar, J. et al. Codon-specific *KRAS* mutations predict survival benefit of trifluridine/tipiracil in metastatic colorectal cancer. *Nat. Med.* **29**, 605–614 (2023). 36864254 10.1038/s41591-023-02240-8 PMC10033412

[48] Academy of Medical Royal Colleges. *Principles for the Implementation of Genomic Medicine* (2019); www.aomrc.org.uk/wp-content/uploads/2019/10/Principles_implementation_genomic_medicine_011019.pdf

[49] Malone, E. R., Oliva, M., Sabatini, P. J. B., Stockley, T. L. & Siu, L. L. Molecular profiling for precision cancer therapies. *Genome Med.* **12**, 8 (2020). 31937368 10.1186/s13073-019-0703-1 PMC6961404

[50] Kato, S. et al. Real-world data from a molecular tumor board demonstrates improved outcomes with a precision N-of-One strategy. *Nat. Commun.* **11**, 4965 (2020). 33009371 10.1038/s41467-020-18613-3 PMC7532150

[51] DETERMINE Precision Medicine. *Cancer Research UK* www.cancerresearchuk.org/funding-for-researchers/our-research-infrastructure/our-centre-for-drug-development/determine-overview (undated).

[52] Cornish, A. J. et al. Whole genome sequencing of 2,023 colorectal cancers reveals mutational landscapes, new driver genes and immune interactions. Preprint at *bioRxiv*10.1101/2022.11.16.515599 (2022).

[53] Degasperi, A. et al. Substitution mutational signatures in whole-genome-sequenced cancers in the UK population. *Science* **376**, science.abl9283 (2022). 35949260 10.1126/science.abl9283 PMC7613262

[54] Prendergast, S. C. et al. Sarcoma and the 100,000 Genomes Project: our experience and changes to practice. *J. Pathol. Clin. Res.* **6**, 297–307 (2020). 32573957 10.1002/cjp2.174 PMC7578291

[55] Trotman, J. et al. The NHS England 100,000 Genomes Project: feasibility and utility of centralised genome sequencing for children with cancer. *Br. J. Cancer* **127**, 137–144 (2022). 35449451 10.1038/s41416-022-01788-5 PMC9276782

[56] Cornish, A. J. et al. Reference bias in the Illumina Isaac aligner. *Bioinformatics* **36**, 4671–4672 (2020). 32437525 10.1093/bioinformatics/btaa514 PMC7653636

[57] Alexandrov, L. B. et al. The repertoire of mutational signatures in human cancer. *Nature* **578**, 94–101 (2020). 32025018 10.1038/s41586-020-1943-3 PMC7054213

[58] How Your Data is Used*. Genomics England* www.genomicsengland.co.uk/patients-participants/data (2023).

[59] 100,000 Genomes Project Bioresource—Main Phase. *NHS Health Research Authority* www.hra.nhs.uk/planning-and-improving-research/application-summaries/research-summaries/100000-genomes-project-bioresource-main-phase (2023).

