# Supplementary material for: Insights for precision oncology from the integration of genomic and clinical data of 13,880 tumors from the 100,000 Genomes Cancer Programme
Source: Nat Med. 2024 Jan 11;30(1):279–89. doi: 10.1038/s41591-023-02682-0 (PMC10803271; doi:10.1038/s41591-023-02682-0)
Supplement: Supplementary file 1 — Anonymized whole-genome analysis results for two participants. Bioinformatics pipeline validation. Cancer report, September 2018. [file 41591_2023_2682_MOESM1_ESM.pdf]

nature medicine

Analysis https://doi.org/10.1038/s41591-023-02682-0

Insights for precision oncology from the integration of genomic and clinical data of 13,880 tumors from the 100,000 Genomes Cancer Programme

In the format provided by the authors and unedited

Anonymised Whole Genome Analysis, Participant 1

Note: The result has been truncated for illustrative purposes to show only the subset of clinically relevant variants and pan-genomic markers.

Whole Genome Analysis

100,000 Genomes Project Cancer Programme

Supplementary analysis: somatic variants and pertinent germline findings in cancer susceptibility genes v1.11

Variants present in the germline are subtracted to produce a list of somatic variants. Accordingly, variants detected in both the germline and the tumour will not be listed in this analysis with the exception of known pathogenic or likely pathogenic variants in cancer susceptibility genes relevant to the tumour type.
The summary of findings provided by Genomics England is accredited under the ISO 15189 standard according to the schedule of accreditation https://www.ukas.com/wp-content/uploads/schedule_uploads/00007/10170Medical%20Single.pdf

Participant information

Participant name	D.O.B.	Gender	NHS number	Laboratory sample ID	Participant ID	GMC	Sample date	Date analysis issued
		FEMALE					06-06-2018	17-08-2019

Tumour information

Disease type	Disease subtype	Annotation group	Tumour Type	Topography	Morphology	Sample type and Library type	Reported tumour content	Tumour sample cross-contamination
OVARIAN	HIGH GRADE SEROUS CARCINOMA	SOLID	PRIMARY	88930	8460/3	FF PCR-free	Medium 40-60%	Pass

Sequencing quality information

See online Technical Information v1.11.main document and/or LabKey QC portal for details and expected ranges of QC metrics

Sample type	Mapped reads, %	Chimeric DNA fragments, %	Insert size median, bp	Genome-wide coverage mean, x	Unevenness of local genome coverage, x	COSMIC content with low coverage (<30x), %	Total somatic SNVs	Total somatic indels	Total somatic SVs
Germline	92.91	0.24	467.0	32.76	7.01	N/A	N/A	N/A	N/A
Tumour	92.71	0.26	492.0	103.72	15.87	0.88	14635	2226	460

PART ONE: DESCRIPTION OF SOMATIC VARIANTS

Circos plot: genome-wide visualisation of somatic variants and sequencing depth

This plot illustrates the distribution of somatic variants across the genome with each concentric circle (track) representing a different class of variant.
Chromosomes are arranged sequentially around the circumference as indicated. The information presented in each track is as follows:

Track 1 (innermost track): chromosomes

Track 2 (in red): number of somatic SNVs in 2Mb window; scale from 0 to 100

Track 3 (in green): number of somatic indels in 2Mb window; scale from 0 to 35

Track 4: ratio of normalised depth of coverage for tumour vs normal in log2 scale smoothed over 100 kb windows. Diploid regions have value of 0. Scale is between -2 and 2. Regions with coverage below 15x in germline are not shown. CNV losses are indicated in red, CNV gains are indicated in green, copy-neutral LOH regions are indicated in yellow.

Track 5 (outermost track, in blue): absolute depth of coverage in tumour sample

Structural variants (SVs) are indicated by arcs inside the plot; translocations are indicated in green, inversions are indicated in purple. SVs shorter than 100 kb and insertions are not plotted.

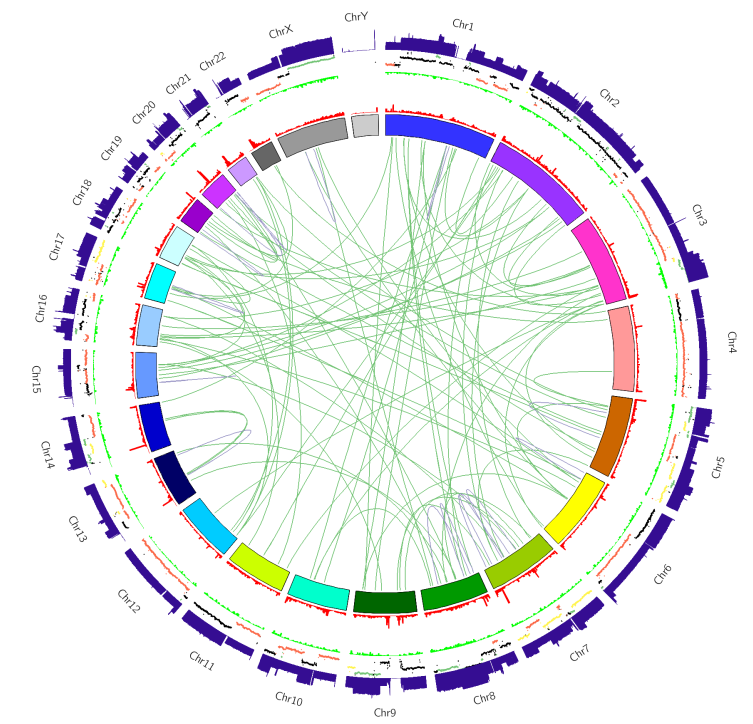

Anonymised Whole Genome Analysis, Participant 1

Small somatic variants detected

Only variants with specific consequences (transcript ablation, splice acceptor variant, splice donor variant, stop gained, frameshift variant, stop lost, start lost, transcript amplification, inframe insertion, inframe deletion, inframe variant, missense variant, splice region variant) in canonical transcripts are reported. Complex indels and frameshift variants are only annotated at the CDS level owing to problems accurately annotating the protein change with the current pipeline. The complete list of canonical transcripts can be accessed at List of canonical transcripts v1.11. Small variants are classified as SNVs and indels <50bp. Classification for gene mode of action (oncogene, tumour suppressor or both) was extracted from the manually curated list of Cancer Census Genes (see below).
Reported variants are classified into three domains:

Domain 1 variants

Variants in a virtual panel of potentially actionable genes*. Actionable genes are defined as genes in which small variants (SNVs and indels <50bp) have reported therapeutic, prognostic or clinical trial associations**, as defined by the GenomOncology Knowledge Management System. For haematological malignancies the information displayed relates to either lymphoid tumours (where the referred tumour type is lymphoid) myeloid tumours (where the referred tumour type is myeloid) or all haematological tumours i.e. lymphoid and myeloid (in the unusual case that the referred tumour type has features of both e.g. biphenotypic leukaemia). Where known, the 'variant-level actionability' category and applicable tumour type are indicated. For other variants in these genes, their impact on gene function has not yet been characterised and therefore their actionability status is unclear. This means:
(i) local evaluation will be required for listed variants which are not yet characterised
(ii) even if well characterised as actionable for some tumour types, the listed variants may not be actionable in the participant's specific tumour type
*Current potentially actionable genes: 143 genes, listed at Actionable genes v1.11 document
**Links are provided to clinical trials within the United Kingdom which are both actively recruiting participants or closed to recruitment.

Gene	GRCh38 coordinates ref/alt allele	Transcript	CDS change and protein change	Predicted consequences	Population germline allele frequency (1KG | gnomAD)	VAF	Alt allele/total read depth	COSMIC ID	Gene-level actionability	Variant-level actionability	Gene mode of action
TP53	17:7674229 C>T	ENST00000269305	c.734G>A p.(Gly245Asp)	missense_variant	- | <0.00005	0.79	42/53	COSM1640832 COSM179807 COSM179806 COSM179805 COSM43606 COSM3388189	Trial (ovarian ca) Trial (NSC lung ca, SCLC, breast ca, ca) Trial (NSC lung ca, ca, colorectal ca, head neck SCC, ovarian ca, prostate ca) Trial (NSC lung ca, breast ca, colorectal ca, esophageal SCC, head neck SCC, ovarian ca, pancreatic ca, urothelial ca) Trial (ovarian serous ca)		both

Complex indels are only annotated at the CDS level

Symbol next to *Predicted consequences* denotes:
(H) an indel intersecting with reference homopolymers of at least 8 nucleotides in length
(N) an indel in the regions with high levels of sequencing noise where at least 10% of the basecalls in a window extending 50 bases to either side of the indel's call have been filtered out due to the poor quality
(G) a variant with a germline allele frequency > 1% in an internal Genomics England data set (indicates potential un-subtracted germline variant)
(R) a recurrently identified somatic variant with somatic allele frequency > 5% in an internal Genomics England data set (indicates potential technical artefact)
(SR) a variant overlapping simple repeats

Symbol next to *VAF* denotes:
(LOH) a small variant that overlaps with copy-neutral loss of heterozygosity region

Domain 2 variants

Variants in a virtual panel of cancer-related genes***. Cancer-related genes are defined as genes in which any variants have been causally implicated in cancer, as defined by the Cancer Gene Census (Wellcome Trust Sanger Institute)
***Current cancer-related genes: 566 genes, listed at Cancer census genes v1.11 document

Gene	GRCh38 coordinates ref/alt allele	Transcript	CDS change and protein change	Predicted consequences	Population germline allele frequency (1KG | gnomAD)	VAF	Alt allele/total read depth	COSMIC ID	Gene mode of action
...	...	...	...	...	...	...	...	...	...

Complex indels are only annotated at the CDS level

Symbol next to *Predicted consequences* denotes:
(H) an indel intersecting with reference homopolymers of at least 8 nucleotides in length
(N) an indel in the regions with high levels of sequencing noise where at least 10% of the basecalls in a window extending 50 bases to either side of the indel's call have been filtered out due to the poor quality
(G) a variant with a germline allele frequency > 1% in an internal Genomics England data set (indicates potential un-subtracted germline variant)
(R) a recurrently identified somatic variant with somatic allele frequency > 5% in an internal Genomics England data set (indicates potential technical artefact)
(SR) a variant overlapping simple repeats

Symbol next to *VAF* denotes:
(LOH) a small variant that overlaps with copy-neutral loss of heterozygosity region

Domain 3 variants

Gene	GRCh38 coordinates ref/alt allele	Transcript	CDS change and protein change	Predicted consequences	Population germline allele frequency (1KG | gnomAD)	VAF	Alt allele/total read depth	COSMIC ID
...	...	...	...	...	...	...	...	...

Complex indels are only annotated at the CDS level

Symbol next to *Predicted consequences* denotes:
(H) an indel intersecting with reference homopolymers of at least 8 nucleotides in length
(N) an indel in the regions with high levels of sequencing noise where at least 10% of the basecalls in a window extending 50 bases to either side of the indel's call have been filtered out due to the poor quality
(G) a variant with a germline allele frequency > 1% in an internal Genomics England data set (indicates potential un-subtracted germline variant)
(R) a recurrently identified somatic variant with somatic allele frequency > 5% in an internal Genomics England data set (indicates potential technical artefact)
(SR) a variant overlapping simple repeats

Symbol next to *VAF* denotes:
(LOH) a small variant that overlaps with copy-neutral loss of heterozygosity region

Structural variants detected

For details of the algorithms used to call CNVs and SVs please refer to Technical Information v1.11.main. Somatic CNVs and SVs variants have not been assigned to domains whilst the performance (recall and precision) of the calling algorithm for CNVs and SVs is under evaluation.
Only SVs overlapping breakends with introns or exons are listed in the table below. Each row corresponds to one structural variant. Types of structural variants called by Canvas: GAIN(COPY NUMBER) = CNV gain, LOSS(COPY NUMBER) = CNV loss, LOH(COPY NUMBER) = loss of heterozygosity. Types of structural variants called by Manta: BND = translocation, DEL = deletion, DUP = duplication, INV = inversion, INS = insertion. Coordinate for the second breakend in translocation event captures replacement string, position and direction according to variant call format specification v4.3

Gene-centered view of structural variants

Gene	Transcript	Genomic coordinates	Type	Size	Cytological bands	CGC	Role in cancer
BRCA1	ENST00000357654	17:39154813-46087911	LOH(2)	6933098	del(17)(q12;q21.31)	CGC	tumour suppressor
BRCA2	ENST00000544455	13:30701479-46497932	LOSS(1)	15796453	del(13)(q12.3;q14.13)	CGC	tumour suppressor
TP53	ENST00000269305	17:5670099-9362302	LOSS(1)	3692203	del(17)(p13.2;p13.1)	CGC	oncogene/tumour suppressor

Anonymised Whole Genome Analysis, Participant 1

PART TWO: ANALYSIS OF SOMATIC VARIANTS

Somatic mutation prevalence (global mutation burden)

Total number of somatic non-synonymous small variants per megabase (coding region): 4.39

The vertical axis (log scaled) shows the number of somatic non-synonymous small variants per megabase of coding sequences. The dashed horizontal red line represents the total somatic mutation burden in this patient's genome. Each sample in the 100,000 Genomes Cancer dataset is represented by a dot on the plot; different cancer types are ordered on the horizontal axis based on their median numbers of somatic mutations (short horizontal red line). Only samples processed with PCR-Free library prep kit and tumour sample cross-contamination < 1% in the 100,000 Genomes Cancer dataset are shown.

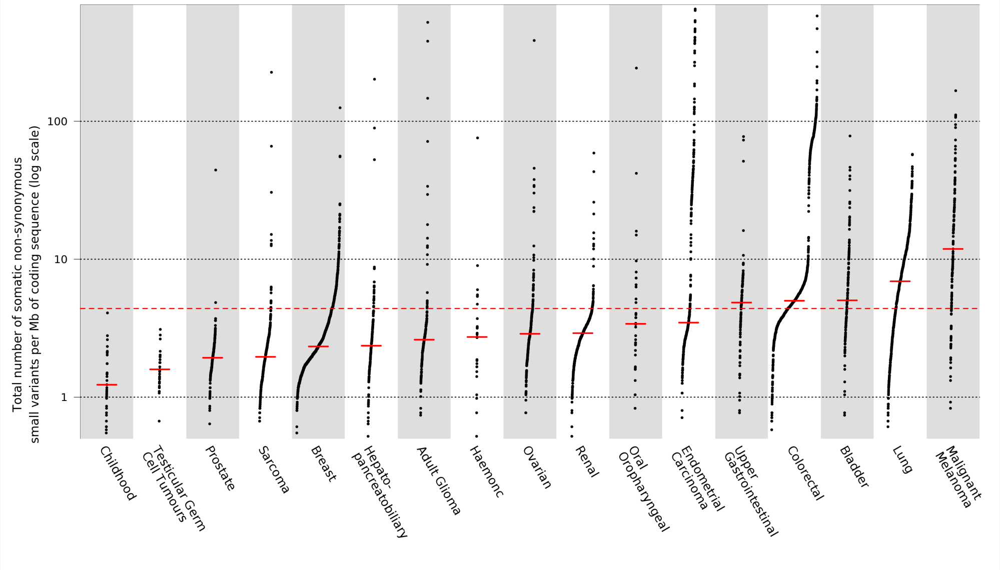

Contextual analysis of somatic SNVs

The following histogram represents the contextual frequency of each type of base substitution. The counts of each mutation-type (i.e. base substitution) at each mutation context (i.e. base situated immediately 3' and 5' to the mutated nucleotide) are normalised by the total number of tri-nucleotide counts in the reference genome. All substitutions are referred to by the pyrimidine context of the mutated base pair. Mutation types are given on the horizontal axis while the percentage of mutations attributed to a specific mutation type are on the vertical axis.

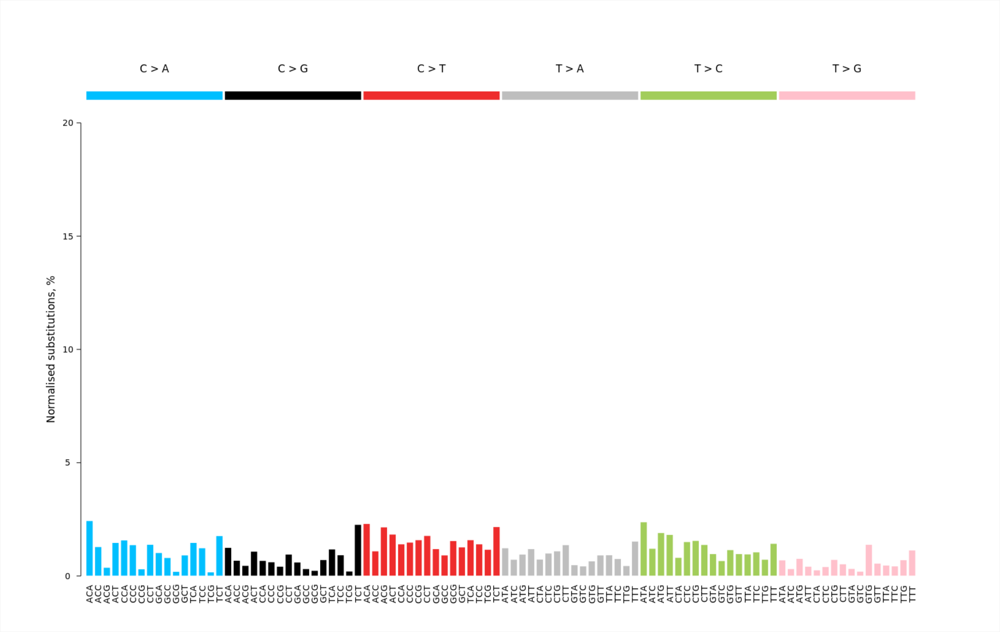

Anonymised Whole Genome Analysis, Participant 1

Mutational signature analysis

The following bar plot displays the relative proportions of the different mutational signatures demonstrated by the tumour. Analysis of large sequencing datasets (10,952 exomes and 1,048 whole-genomes from 40 distinct tumour types) has allowed patterns of relative contextual frequencies of different SNVs to be grouped into specific mutational signatures. Using mathematical methods (decomposition by non-negative least squares) the contribution of each of these signatures to the overall mutation burden observed in a tumour can be derived. Further details of the 30 different mutational signatures used for this analysis, their prevalence in different tumour types and proposed aetiology can be found at the [Sanger Institute Website](#).

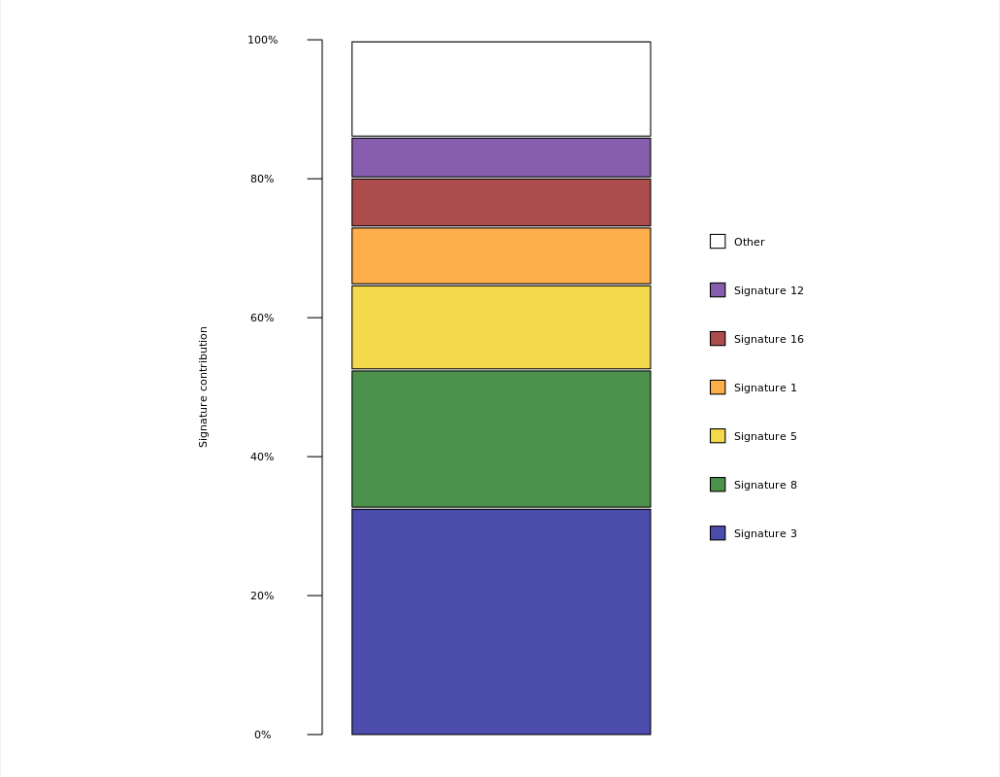

Deviance (residual sum of squares) between fitted data and actual data is 0.0005

Analysis of clusters of somatic SNVs (rain plot)

This plot presents SNVs in positional order (from the first variant on the short arm of chromosome 1 to the last variant on the long arm of chromosome Y) on the X-axis with the distance between consecutive SNVs in logarithmic scale indicated on the Y-axis. The colour of each dot indicates the type of substitution (see legend). Regions of localized hypermutation can be observed as clusters of SNVs that have lower inter-mutation distance and show similar base changes.
Putative regions of hypermutation are indicated by black arrows on this plot and have been determined as regions containing six or more consecutive mutations with an average intermutation distance of less than or equal to 1,000 bp.

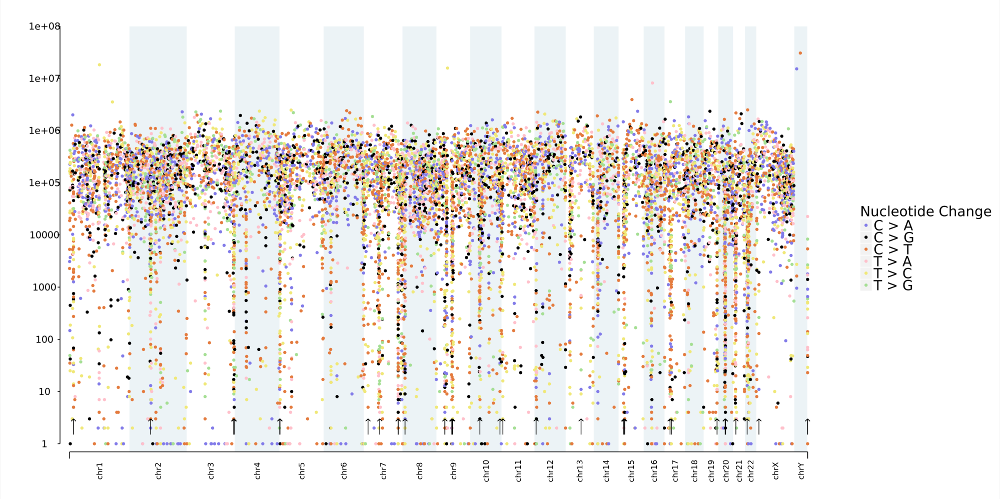

The table below indicates the genomic coordinates of individual regions of hypermutation.
[Click to collapse/expand.](#)

Anonymised Whole Genome Analysis, Participant 1

Analysis of variant allele frequency (VAF) of small somatic variants and indel lengths

The following histograms show VAF and length distributions for small somatic variants.
VAF is calculated as alt/(alt + ref) where alt and ref are the number of reads passing filter (see Technical Information v1.11.main) supporting the non-reference and reference base respectively. VAF depends on tumour purity, cancer heterogeneity and copy number variants.

The negative values on the length distribution plot correspond to deletions and the positive values correspond to insertions

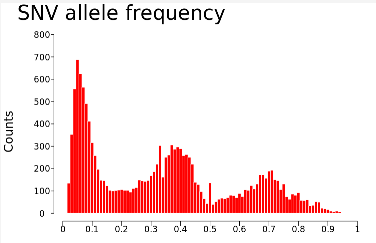

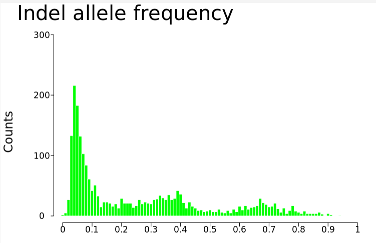

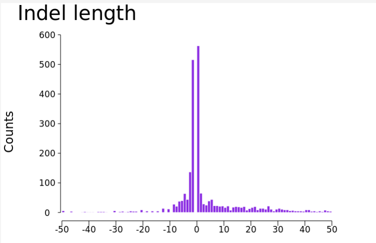

PART THREE: ANALYSIS OF GERMLINE VARIANTS

Germline analysis: variants in cancer susceptibility genes

Tier 1 includes variants deemed to be pathogenic or likely pathogenic which are in cancer susceptibility genes relevant to the disease type. It is recommended that clinical review and confirmation of these findings are undertaken locally as appropriate. The genes included are available from PanelApp - Ovarian cancer pertinent cancer susceptibility (Version 1.0).

Tier 3 contains all rare variants arising across a large set of cancer susceptibility genes. Review of Tier 3 germline variants is not required routinely but should be considered in cases in which there is a high index of suspicion of a germline determinant of cancer in the patient and/or family. The genes included are available from PanelApp - Adult solid tumours cancer susceptibility (Version 1.7).

For more details of analysis please refer to Technical Information v1.11.main.

Tier 1 variants

Gene	GRCh38 coordinates ref/alt allele	Transcript	CDS change and protein change	Predicted consequences	Population germline allele frequency (1KG | gnomAD)	Genomics England germline allele frequency	Alt allele/total read depth	Genotype	ClinVar ID	ClinVar data	Gene mode of action
BRCA1	17:43124027 ACT>A	ENST00000357654	c.68_69delAG	frameshift_variant	- | 0.0002	.	24/44	0/1	17662	★★★☆ Pathogenic	tumor suppressor gene

Symbol next to *Predicted consequences* denotes:
(T) the variant is in the final 10% of the coding region and the impact of protein truncation regarding pathogencity should be evaluated

Tier 3 variants

No pertinent germline findings detected in tier 3

Genes marked with an asterisk (*) are in the panel for the patient's disease type

Symbol next to *Predicted consequences* denotes:
(T) the variant is in the final 10% of the coding region and the impact of protein truncation regarding pathogencity should be evaluated

Germline analysis: Pharmacogenomics

Polymorphic variants in the DPYD gene are associated with severe, sometimes fatal, toxicity to fluoropyrimidine therapy. Analysis was undertaken for 4 established major variants/haplotypes in DPYD (see Technical Information v1.11.main). The following toxicity-related DPYD variant(s) were detected.

No toxicity-related DPYD variants were detected.

Additional rarer DPYD variants are associated with toxicity on fluoropyrimidine therapy. Additional DPYD haplotypes are associated with toxicity on fluoropyrimidine therapy that is less severe. Other genetic, clinical and environmental factors may affect a patient's response to fluoropyrimidines and their risk for adverse drug reactions. Chromosomal phasing has not been performed. Interpretation recommendations are based on the assumption that when two variants relating to reduced function are detected, they are in trans (on different chromosomes).

This is a research result. We are returning this to help clinical teams reduce risk of harm from medications. If the result is intended for use in informing clinical management it should be confirmed using a test accredited for clinical use. It remains the responsibility of the health-care provider to determine the best course of treatment for a patient.

Additional Information

- **Sensitivity: the depth of WGS used in this analysis will typically detect 99% of somatic SNVs with an allele frequency of ≥0.3, 95% of somatic SNVs with an allele frequency of ≥0.1 and 60% of somatic small indels (<50bp) with an allele frequency of ≥0.2 (estimate is based upon admixtures analysis of a highly accurate catalog of variants produced in the 'platinum genomes' project). Consequently, somatic variants with allelic frequencies below this level, or in areas of low coverage may not be detected. The sensitivity for detection of SVs and CNVs is yet to be determined. False negative results cannot be excluded.**
- Specificity: as yet the expected false positive rates across a range of somatic variant types and allele frequencies has not been determined. Therefore false positive results cannot be excluded.
- Variant calls are filtered according to the quality and quantity of reads. Full details of the filters used in this analysis can be found in the Technical Information v1.11.main.
- In this analysis MNVs (multiple nucleotide variants) can be reported as multiple consecutive SNVs and therefore the protein change may require correction.
- A somatic variant may have multiple entries in COSMIC database due to the use of different reference sequences. In these cases links to all COSMIC entries are provided.
- The germline analysis undertaken may not be fully sensitive on account of coverage and the reference data used for assessment of non-truncating variants. If the patient has been evaluated as clinically eligible for germline genetic testing on account of their personal and/or family history of cancer, this testing should be performed as per standard local practice.
- If a pathogenic or likely pathogenic germline susceptibility variant is detected, it is recommended that the variant is reviewed by a local clinical laboratory service with expertise in germline cancer genetics. Referral to a clinical cancer genetics unit and technical confirmation of the variant in a new blood sample may be recommended following local variant review.
- For a full description of the methods used to produce these results and for further information regarding QC metrics please refer to the Technical Information v1.11.main. All related documentation is available at Genomics England Website.
- 'N/A' indicates that information is not available or not applicable.

Anonymised Whole Genome Analysis, Participant 2

Note: The result has been truncated for illustrative purposes to show only the subset of clinically relevant variants and pan-genomic markers.

Whole Genome Analysis

100,000 Genomes Project Cancer Programme

Supplementary analysis: somatic variants and pertinent germline findings in cancer susceptibility genes v1.11

Genomics england

UKAS MEDICAL 10170

Variants present in the germline are subtracted to produce a list of somatic variants. Accordingly, variants detected in both the germline and the tumour will not be listed in this analysis with the exception of known pathogenic or likely pathogenic variants in cancer susceptibility genes relevant to the tumour type.
The summary of findings provided by Genomics England is accredited under the ISO 15189 standard according to the schedule of accreditation https://www.ukas.com/wp-content/uploads/schedule_uploads/00007/10170Medical%20Single.pdf

Participant information

Participant name	D.O.B.	Gender	NHS number	Laboratory sample ID	Participant ID	GMC	Sample date	Date analysis issued
		FEMALE					06-06-2018	26-05-2019

Tumour information

Disease type	Disease subtype	Annotation group	Tumour Type	Topography	Morphology	Sample type and Library type	Reported tumour content	Tumour sample cross-contamination
ENDOMETRIAL_CARCINOMA	ENDOMETRIOID ADENOCARCINOMA	SOLID	PRIMARY	2739003	30289006	FF PCR-free	High >60%	Pass

Sequencing quality information

See online Technical Information v1.11.main document and/or LabKey QC portal for details and expected ranges of QC metrics

Sample type	Mapped reads, %	Chimeric DNA fragments, %	Insert size median, bp	Genome-wide coverage mean, x	Unevenness of local genome coverage, x	COSMIC content with low coverage (<30x), %	Total somatic SNVs	Total somatic indels	Total somatic SVs
Germline	92.9	0.28	456.8	29.55	6.83	N/A	N/A	N/A	N/A
Tumour	94.2	0.42	453.0	112.00	16.59	0.57	79419	209436	133

PART ONE: DESCRIPTION OF SOMATIC VARIANTS

Circos plot: genome-wide visualisation of somatic variants and sequencing depth

This plot illustrates the distribution of somatic variants across the genome with each concentric circle (track) representing a different class of variant. Chromosomes are arranged sequentially around the circumference as indicated. The information presented in each track is as follows:

Track 1 (innermost track): chromosomes

Track 2 (in red): number of somatic SNVs in 2Mb window; scale from 0 to 100

Track 3 (in green): number of somatic indels in 2Mb window; scale from 0 to 35

Track 4: ratio of normalised depth of coverage for tumour vs normal in log2 scale smoothed over 100 kb windows. Diploid regions have value of 0. Scale is between -2 and 2. Regions with coverage below 15x in germline are not shown. CNV losses are indicated in red, CNV gains are indicated in green, copy-neutral LOH regions are indicated in yellow.

Track 5 (outermost track, in blue): absolute depth of coverage in tumour sample

Structural variants (SVs) are indicated by arcs inside the plot; translocations are indicated in green, inversions are indicated in purple. SVs shorter than 100 kb and insertions are not plotted.

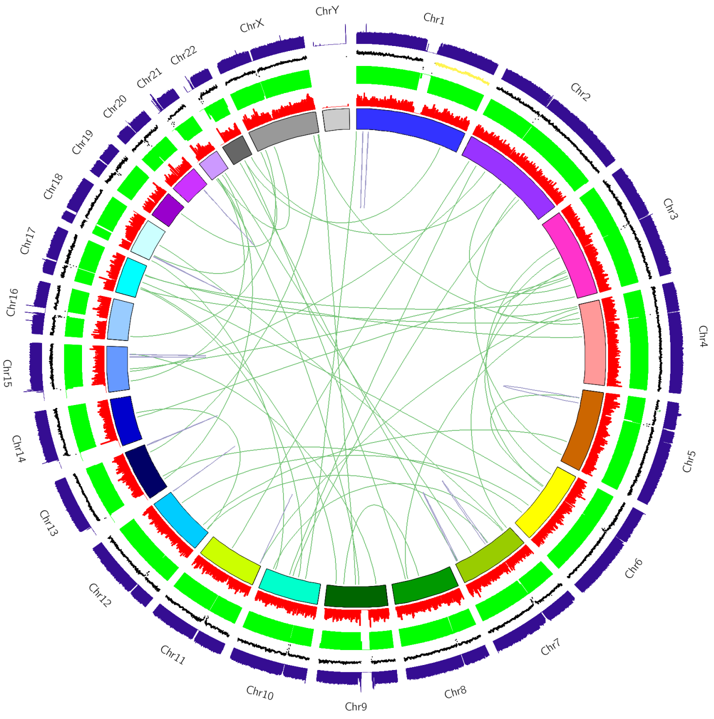

Anonymised Whole Genome Analysis, Participant 2

Small somatic variants detected

Only variants with specific consequences (transcript ablation, splice acceptor variant, splice donor variant, stop gained, frameshift variant, stop lost, start lost, transcript amplification, inframe insertion, inframe deletion, inframe variant, missense variant, splice region variant) in canonical transcripts are reported. Complex indels and frameshift variants are only annotated at the CDS level owing to problems accurately annotating the protein change with the current pipeline. The complete list of canonical transcripts can be accessed at List of canonical transcripts v1.11. Small variants are classified as SNVs and indels <50bp. Classification for gene mode of action (oncogene, tumour suppressor or both) was extracted from the manually curated list of Cancer Census Genes (see below).
Reported variants are classified into three domains:

Domain 1 variants

Variants in a virtual panel of potentially actionable genes*. Actionable genes are defined as genes in which small variants (SNVs and indels <50bp) have reported therapeutic, prognostic or clinical trial associations**, as defined by the GenomOncology Knowledge Management System. For haematological malignancies the information displayed relates to either lymphoid tumours (where the referred tumour type is lymphoid) myeloid tumours (where the referred tumour type is myeloid) or all haematological tumours i.e. lymphoid and myeloid (in the unusual case that the referred tumour type has features of both e.g. biphenotypic leukaemia). Where known, the 'variant-level actionability' category and applicable tumour type are indicated. For other variants in these genes, their impact on gene function has not yet been characterised and therefore their actionability status is unclear. This means:
(i) local evaluation will be required for listed variants which are not yet characterised
(ii) even if well characterised as actionable for some tumour types, the listed variants may not be actionable in the participant's specific tumour type
*Current potentially actionable genes: 143 genes, listed at Actionable genes v1.11 document
**Links are provided to clinical trials within the United Kingdom which are both actively recruiting participants or closed to recruitment.

Gene	GRCh38 coordinates ref/alt allele	Transcript	CDS change and protein change	Predicted consequences	Population germline allele frequency (1KG | gnomAD)	VAF	Alt allele/total read depth	COSMIC ID	Gene-level actionability	Variant-level actionability	Gene mode of action
PMS2	7:6009019 T>C	ENST00000265849	c.1A>G p.(Met1?)	start_lost	- | <0.00005	0.28	29/104	N/A	Trial (NSC lung ca, RCC, SCLC, bladder ca, colorectal ca, esophageal ca, gastric ca, melanoma) Trial (SCC Lung, SCLC, ca, cervical ca, colorectal ca, endometrial ca, lung ca, ovarian ca, prostate ca)		N/A

Complex indels are only annotated at the CDS level

Symbol next to *Predicted consequences* denotes:
(H) an indel intersecting with reference homopolymers of at least 8 nucleotides in length
(N) an indel in the regions with high levels of sequencing noise where at least 10% of the basecalls in a window extending 50 bases to either side of the indel's call have been filtered out due to the poor quality
(G) a variant with a germline allele frequency > 1% in an internal Genomics England data set (indicates potential un-subtracted germline variant)
(R) a recurrently identified somatic variant with somatic allele frequency > 5% in an internal Genomics England data set (indicates potential technical artefact)
(SR) a variant overlapping simple repeats

Symbol next to *VAF* denotes:
(LOH) a small variant that overlaps with copy-neutral loss of heterozygosity region

Domain 2 variants

Variants in a virtual panel of cancer-related genes***. Cancer-related genes are defined as genes in which any variants have been causally implicated in cancer, as defined by the Cancer Gene Census (Wellcome Trust Sanger Institute)
***Current cancer-related genes: 566 genes, listed at Cancer census genes v1.11 document

Gene	GRCh38 coordinates ref/alt allele	Transcript	CDS change and protein change	Predicted consequences	Population germline allele frequency (1KG | gnomAD)	VAF	Alt allele/total read depth	COSMIC ID	Gene mode of action
...	...	...	...	...	...	...	...	...	...

Complex indels are only annotated at the CDS level

Symbol next to *Predicted consequences* denotes:
(H) an indel intersecting with reference homopolymers of at least 8 nucleotides in length
(N) an indel in the regions with high levels of sequencing noise where at least 10% of the basecalls in a window extending 50 bases to either side of the indel's call have been filtered out due to the poor quality
(G) a variant with a germline allele frequency > 1% in an internal Genomics England data set (indicates potential un-subtracted germline variant)
(R) a recurrently identified somatic variant with somatic allele frequency > 5% in an internal Genomics England data set (indicates potential technical artefact)
(SR) a variant overlapping simple repeats

Symbol next to *VAF* denotes:
(LOH) a small variant that overlaps with copy-neutral loss of heterozygosity region

Domain 3 variants

Gene	GRCh38 coordinates ref/alt allele	Transcript	CDS change and protein change	Predicted consequences	Population germline allele frequency (1KG | gnomAD)	VAF	Alt allele/total read depth	COSMIC ID
...	...	...	...	...	...	...	...	...

Complex indels are only annotated at the CDS level

Symbol next to *Predicted consequences* denotes:
(H) an indel intersecting with reference homopolymers of at least 8 nucleotides in length
(N) an indel in the regions with high levels of sequencing noise where at least 10% of the basecalls in a window extending 50 bases to either side of the indel's call have been filtered out due to the poor quality
(G) a variant with a germline allele frequency > 1% in an internal Genomics England data set (indicates potential un-subtracted germline variant)
(R) a recurrently identified somatic variant with somatic allele frequency > 5% in an internal Genomics England data set (indicates potential technical artefact)
(SR) a variant overlapping simple repeats

Symbol next to *VAF* denotes:
(LOH) a small variant that overlaps with copy-neutral loss of heterozygosity region

Structural variants detected

For details of the algorithms used to call CNVs and SVs please refer to Technical Information v1.11.main. Somatic CNVs and SVs variants have not been assigned to domains whilst the performance (recall and precision) of the calling algorithm for CNVs and SVs is under evaluation.
Only SVs overlapping breakends with introns or exons are listed in the table below. Each row corresponds to one structural variant. Types of structural variants called by Canvas: GAIN(COPY NUMBER) = CNV gain, LOSS(COPY NUMBER) = CNV loss, LOH(COPY NUMBER) = loss of heterozygosity. Types of structural variants called by Manta: BND = translocation, DEL = deletion, DUP = duplication, INV = inversion, INS = insertion. Coordinate for the second breakend in translocation event captures replacement string, position and direction according to variant call format specification v4.3

Gene-centered view of structural variants

Gene	Transcript	Genomic coordinates	Type	Size	Cytological bands	CGC	Role in cancer
...	...	...	...	...	...	...	...

Anonymised Whole Genome Analysis, Participant 2

PART TWO: ANALYSIS OF SOMATIC VARIANTS

Somatic mutation prevalence (global mutation burden)

Total number of somatic non-synonymous small variants per megabase (coding region): 42.87

The vertical axis (log scaled) shows the number of somatic non-synonymous small variants per megabase of coding sequences. The dashed horizontal red line represents the total somatic mutation burden in this patient's genome. Each sample in the 100,000 Genomes Cancer dataset is represented by a dot on the plot; different cancer types are ordered on the horizontal axis based on their median numbers of somatic mutations (short horizontal red line). Only samples processed with PCR-Free library prep kit and tumour sample cross-contamination < 1% in the 100,000 Genomes Cancer dataset are shown.

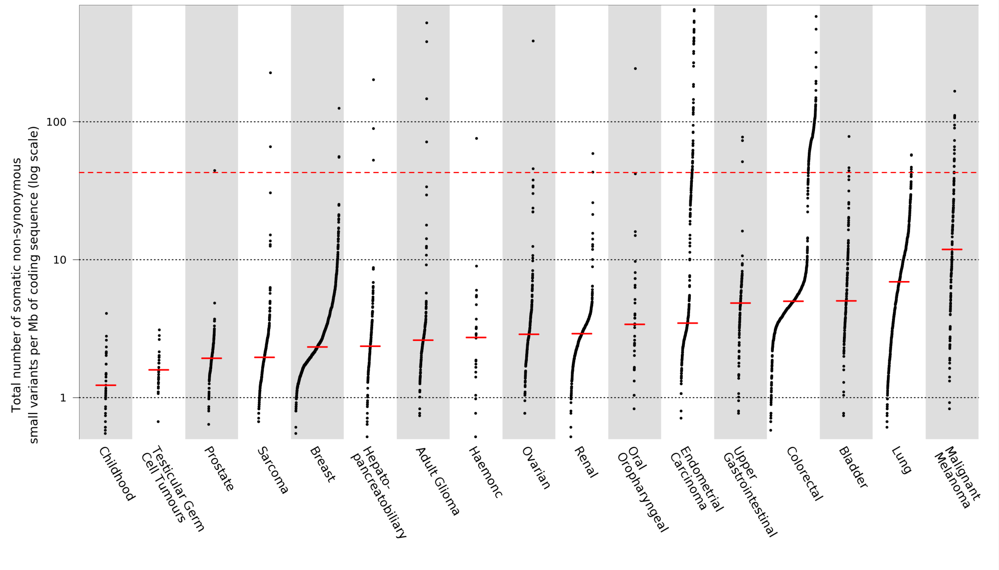

Contextual analysis of somatic SNVs

The following histogram represents the contextual frequency of each type of base substitution. The counts of each mutation-type (i.e. base substitution) at each mutation context (i.e. base situated immediately 3' and 5' to the mutated nucleotide) are normalised by the total number of tri-nucleotide counts in the reference genome. All substitutions are referred to by the pyrimidine context of the mutated base pair. Mutation types are given on the horizontal axis while the percentage of mutations attributed to a specific mutation type are on the vertical axis.

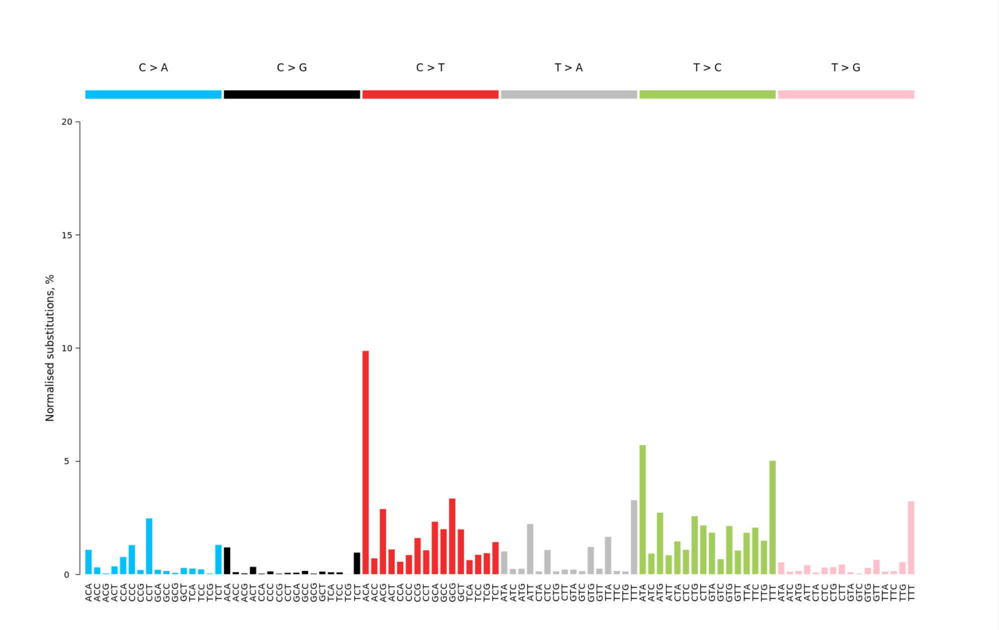

Anonymised Whole Genome Analysis, Participant 2

Mutational signature analysis

The following bar plot displays the relative proportions of the different mutational signatures demonstrated by the tumour. Analysis of large sequencing datasets (10,952 exomes and 1,048 whole-genomes from 40 distinct tumour types) has allowed patterns of relative contextual frequencies of different SNVs to be grouped into specific mutational signatures. Using mathematical methods (decomposition by non-negative least squares) the contribution of each of these signatures to the overall mutation burden observed in a tumour can be derived. Further details of the 30 different mutational signatures used for this analysis, their prevalence in different tumour types and proposed aetiology can be found at the [Sanger Institute Website](#).

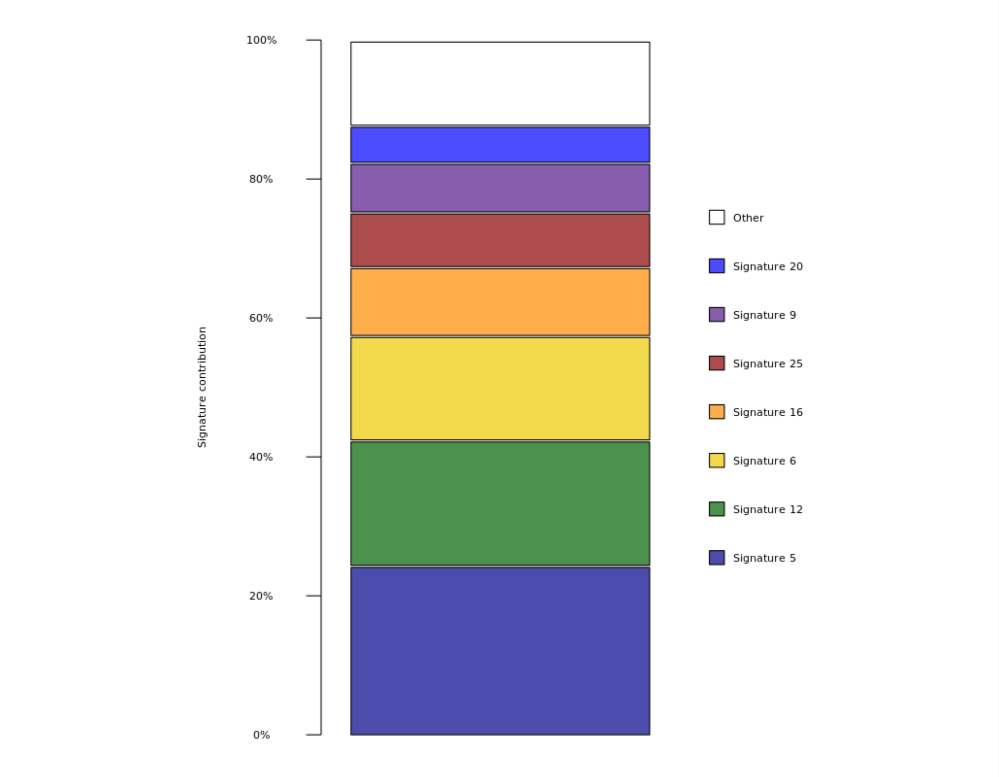

Deviance (residual sum of squares) between fitted data and actual data is 0.0104

Analysis of clusters of somatic SNVs (rain plot)

This plot presents SNVs in positional order (from the first variant on the short arm of chromosome 1 to the last variant on the long arm of chromosome Y) on the X-axis with the distance between consecutive SNVs in logarithmic scale indicated on the Y-axis. The colour of each dot indicates the type of substitution (see legend). Regions of localized hypermutation can be observed as clusters of SNVs that have lower inter-mutation distance and show similar base changes.
Putative regions of hypermutation are indicated by black arrows on this plot and have been determined as regions containing six or more consecutive mutations with an average intermutation distance of less than or equal to 1,000 bp.

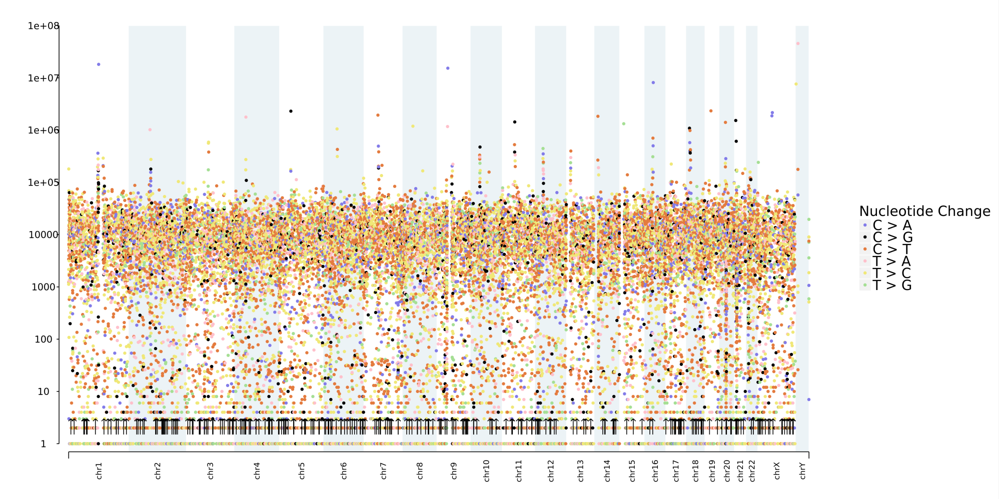

The table below indicates the genomic coordinates of individual regions of hypermutation.
[Click to collapse/expand.](#)

Anonymised Whole Genome Analysis, Participant 2

Analysis of variant allele frequency (VAF) of small somatic variants and indel lengths

The following histograms show VAF and length distributions for small somatic variants.
VAF is calculated as alt/(alt + ref) where alt and ref are the number of reads passing filter (see Technical Information v1.11.main) supporting the non-reference and reference base respectively. VAF depends on tumour purity, cancer heterogeneity and copy number variants.

The negative values on the length distribution plot correspond to deletions and the positive values correspond to insertions

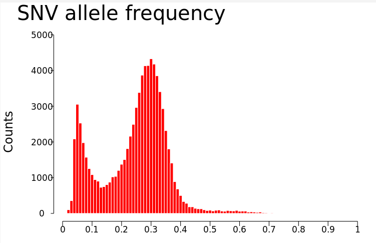

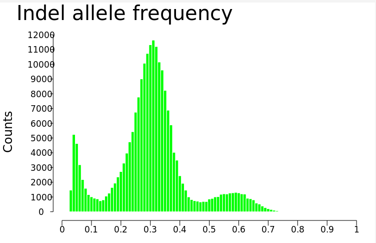

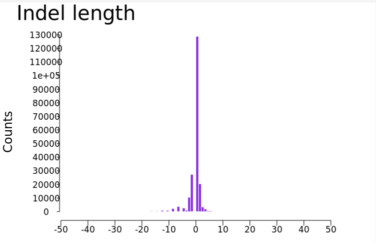

PART THREE: ANALYSIS OF GERMLINE VARIANTS

Germline analysis: variants in cancer susceptibility genes

Tier 1 includes variants deemed to be pathogenic or likely pathogenic which are in cancer susceptibility genes relevant to the tumour type. Clinical review and confirmation of the variant pathogenicity are essential and should be undertaken locally. Technical validation should be performed in an independent sample for any variants that are being reported clinically. The genes included are available from PanelApp - Endometrial cancer pertinent cancer susceptibility (Version 1.0).

Tier 3 contains all rare variants arising across a large set of cancer susceptibility genes. Review of Tier 3 germline variants is not required routinely but should be considered in cases in which there is a high index of suspicion of a germline determinant of cancer in the patient and/or family. The genes included are available from PanelApp - Adult solid tumours cancer susceptibility (Version 1.3).

For more details of analysis please refer to Technical Information v1.11.main.

Tier 1 variants

Gene	GRCh38 coordinates ref/alt allele	Transcript	CDS change and protein change	Predicted consequences	Population germline allele frequency (1KG | gnomAD)	Genomics England germline allele frequency	Alt allele/total read depth	Genotype	ClinVar ID	ClinVar data	Gene mode of action
PMS2	7:5987125 GAA>G	ENST00000265849	c.1638_1639delTT	frameshift_variant	- | -	.	18/33	0/1	216075	★★☆☆ Pathogenic	N/A

Symbol next to *Predicted consequences* denotes:
(T) the variant is in the final 10% of the coding region and the impact of protein truncation regarding pathogencity should be evaluated

Tier 3 variants

Click to collapse/expand

Germline analysis: Pharmacogenomics

Polymorphic variants in the DPYD gene are associated with severe, sometimes fatal, toxicity to fluoropyrimidine therapy. Analysis was undertaken for 4 established major variants/haplotypes in DPYD (see Technical Information v1.11.main). The following toxicity-related DPYD variant(s) were detected.

Gene	GRCh38 coordinates ref/alt allele	Transcript	CDS change and protein change	Predicted consequences	Population germline allele frequency (1KG | gnomAD)	Genomics England germline allele frequency	Alt allele/total read depth	PharmGKB_ID	Genotype
DPYD	1:97450058C>T	ENST00000370192	c.1905+1G>A	splice_donor_variant	0.0063	0.0041	17/35	PA166153760	0/1

‡ Variants c.1129-5923C>G (rs75017182) and c.1236G>A (rs56038477) are part of a haplotype and should not be considered two independent variants for interpretation.

Additional rarer DPYD variants are associated with toxicity on fluoropyrimidine therapy. Additional DPYD haplotypes are associated with toxicity on fluoropyrimidine therapy that is less severe. Other genetic, clinical and environmental factors may affect a patient's response to fluoropyrimidines and their risk for adverse drug reactions. Chromosomal phasing has not been performed. Interpretation recommendations are based on the assumption that when two variants relating to reduced function are detected, they are in trans (on different chromosomes).

This is a research result. We are returning this to help clinical teams reduce risk of harm from medications. If the result is intended for use in informing clinical management it should be confirmed using a test accredited for clinical use. It remains the responsibility of the health-care provider to determine the best course of treatment for a patient.

Additional Information

- **Sensitivity: the depth of WGS used in this analysis will typically detect 99% of somatic SNVs with an allele frequency of ≥0.3, 95% of somatic SNVs with an allele frequency of ≥0.1 and 60% of somatic small indels (<50bp) with an allele frequency of ≥0.2 (estimate is based upon admixtures analysis of a highly accurate catalog of variants produced in the 'platinum genomes' project). Consequently, somatic variants with allelic frequencies below this level, or in areas of low coverage may not be detected. The sensitivity for detection of SVs and CNVs is yet to be determined. False negative results cannot be excluded.**
- Specificity: as yet the expected false positive rates across a range of somatic variant types and allele frequencies has not been determined. Therefore false positive results cannot be excluded.
- Variant calls are filtered according to the quality and quantity of reads. Full details of the filters used in this analysis can be found in the Technical Information v1.11.main.
- In this analysis MNVs (multiple nucleotide variants) can be reported as multiple consecutive SNVs and therefore the protein change may require correction.
- A somatic variant may have multiple entries in COSMIC database due to the use of different reference sequences. In these cases links to all COSMIC entries are provided.
- The germline analysis undertaken may not be fully sensitive on account of coverage and the reference data used for assessment of non-truncating variants. If the patient has been evaluated as clinically eligible for germline genetic testing on account of their personal and/or family history of cancer, this testing should be performed as per standard local practice.
- If a pathogenic or likely pathogenic germline susceptibility variant is detected, it is recommended that the variant is reviewed by a local clinical laboratory service with expertise in germline cancer genetics. Referral to a clinical cancer genetics unit and technical confirmation of the variant in a new blood sample may be recommended following local variant review.
- For a full description of the methods used to produce these results and for further information regarding QC metrics please refer to the Technical Information v1.11.main. All related documentation is available at Genomics England Website.
- 'N/A' indicates that information is not available or not applicable.

Genomics England
1 Canada Square
Canary Wharf
London
E14 5AA

Sequencing Laboratory
Illumina Laboratory Services United Kingdom - Hinxton
The Ogilvie Building, Wellcome Trust Genome Campus
Hinxton Nr Saffron Walden
Essex
CB10 1DR

Bioinformatics Pipeline Validation: Cancer Report, September 2018

Table of Contents

1 Summary **3**
1.1 Somatic Small Variants 3
1.2 Somatic Copy Number Variants (CNVs) ans Structural Variants (SVs) 4
1.3 Overall summary of performance 5

2 Methods **6**
2.1 Validation Sets 6
2.2 Measurements 6
2.3 Output 6

3 Validation Experiment 1: Calling Somatic Small Variants **7**
3.1 Background / Justification 7
3.2 Expected Outcome of Validation 7
3.3 Methods 7
3.3.1 Method Overview 7
3.3.2 Truth set sources 7
3.4 Validation Results 8
3.5 Conclusions 8

4 Validation Experiment 2: Calling Somatic Small Variants **9**
4.1 Background / Justification 9
4.2 Expected Outcome of Validation 9
4.3 Methods 9
4.3.1 Method Overview 9
4.3.2 Truth set sources. 9
4.4 Validation Results 9
4.4.1 Sensitivity 10
4.4.2 Precision 11
4.4.3 Measurement of uncertainty 13
4.5 Limitations 13
4.6 Conclusions 13

5 Validation Experiment 3: Calling Somatic Copy Number Variants and Structural Variants **15**
5.1 Background / Justification 15
5.2 Expected Outcome of Validation 15
5.3 Methods 15
5.3.1 Method Overview 15
5.3.2 Truth set sources 15
5.4 Validation Results 15
5.5 Limitations 16
5.6 Conclusions 17

6 List of Abbreviations **18**

7 References **19**

1 Summary

This document is a summary of the validation and verification of the entire end-to-end cancer bioinformatics pipeline in production that has been run in September 2018 with specific controlled versions of each software module. The document details how the cancer Whole Genome Sequencing (WGS) pipeline is successfully identifying SNVs, Indels and CNVs/SVs in the genomes of cancer patients

Validation framework is based on Guidelines for the Validation of Next Generation Sequencing[1]

1.1 Somatic Small Variants

Illumina bioinformatics team (ISAAC/Strelka developers) have previously provided us with an estimate of sensitivity for ISAAC/Strelka[2,3] pipeline. Sensitivity: at the depths of WGS used in this analysis (75X for tumour and 30X for germline), the test will typically detect 99% of somatic SNVs with an allele frequency of ≥0.3, 95% of somatic SNVs with an allele frequency of ≥0.1 and 60% of somatic indels with an allele frequency of ≥0.2 (estimate is based upon admixtures analysis of a highly accurate catalog of variants produced in the 'platinum genomes' project). Below we present the results of experiments that aim to confirm this statement

The validation design for small variants consisted of two parts. The first part (experiment 1) is based on comparison of results obtained from the Genomics England somatic small variant WGS pipeline and results obtained from three separate clinical diagnostic laboratories using different NGS panel tests. The second part (experiment 2) is based on comparison of the WGS pipeline with high depth exome sequencing.

In the first experiment 96 patients with 157 somatic variants (151 SNVs and 6 indels) detected by panel testing were included in the analysis. WGS detected 155 variants out of the 157 (98.7%) reported by the panel essay. One variant (0.6%) came up exclusively in WGS. All discrepant variants had support < 5% in WGS. There were too few indels included in this experiment to calculate accuracy of indel calling separately. This analysis was focused on a set of well-studied clinically-relevant cancer genes (Domain 1 in Whole Genome Analysis (WGA) results).

Positive percentage agreement, PPAtotal = 155/157 = 98.7%

Positive predictive value, PPVtotal = 155/156 = 99.4%

Note that this analysis assumes that the panel is 100% sensitive and specific. This is unlikely to be true but making this assumption allows us to compare WGS results with standard of care test.

Our second experiment compared results of WGS analysis with a high depth and high confidence exome sequencing data set. This was undertaken not with a 75X/30X genome but rather the standard genome received from Illumina which is 100X/33X. For the 16 samples, the sensitivity for somatic SNVs in the test set with a Variant Allele Frequency (VAF)>10% (as estimated from exome sequencing) was 98% (sample range 93%-100%) from 7986 investigated variant positions. Sensitivity for somatic indels with a VAF>10% (as estimated in exome sequencing) over all 16 samples was 95% (sample range 83%-100%) from 323 investigated variant positions. We observed strong correlation for VAFs calculated from exomes and WGS (n=7953, R2=0.89, p-value < 2.2e-16).

We also attempted to estimate precision using the exome data set although we anticipate that due to extensive filtering of potential False Positives the exome data set can miss True Positive variants and therefore precision can be under-estimated. Our calculations included only non-synonymous variants in protein coding genes (somatic variants in Domains 1-3 in WGA results). We also excluded from this analysis categories of somatic variants that are flagged in WGA results as potential False Positives. Across all 16 samples, the precision for somatic SNVs in the test set with a VAF>10% (as estimated from WGS) was 90% (sample range 57%-96%) from 3666 investigated variant positions. Precision for somatic indels with a VAF>10% (as estimated in WGS) over all 16 samples was 85% (sample range 50%-96%) from 180 investigated variant positions.

To investigate uncertainty we conducted an experiment with variant calling in multiple replicates in the subsets of reads randomly down-sampled from high depth WGS data. Our results have shown that 98% of variants from exome true set with VAF > 10% get consistently recovered with every replicate at 100x tumour depth. This value is not improving with increasing tumour depth. Our experiment for measurement of uncertainty was limited to the variant calling step. We are working on proposal together with Illumina to estimate reproducibility of end-to-end pipeline from re-sequence genomes for cohort of 10 patients.

Based on the results of these two experiments we can conclude that sensitivity of our pipeline for somatic small variants with a VAF >10% is for SNVs > 98% and for indels > 95%. Therefore, the results of our validation have exceeded estimates provided by Illumina (do note that actual coverage is higher than Illumina's evaluation and our analysis was limited to exome regions of the genome) and we consider this part of the pipeline as validated.

All samples in this experiment underwent PCR-free library preparation and cross-patient contamination in tumour samples was < 1%. Therefore, this validation does not cover samples that underwent Nano sequencing library preparation protocol and samples with 1-5% of contamination (these samples are flagged in WGA results sent to Genomic Medicine Center (GMC); results for samples with > 5% contamination are not returned to GMC and replacing samples are requested by Sequencing team)

1.2 Somatic Copy Number Variants (CNVs) ans Structural Variants (SVs)

This experiment is based on the comparison of results obtained from the Genomics England somatic SVs/CNVs WGS pipeline and results obtained from three separate clinical diagnostic laboratories using different Fluorescence in situ hybridization (FISH) tests on haematological tumours. 35 patients with results of 254 standard of care FISH tests (138 SVs (Fusions or other Rearrangements) - 18 positive and 120 negative; and 116 CNVs (Gains or Losses) - 86 positive and 30 negative) were included in the analysis. WGS confirmed all positive findings for SVs. Four SVs that come up negative on FISH test were detected by WGS with high confidence. Computational variant calling for CNVs was supplemented by visual inspection of coverage for specific chromosomal regions on circos plot. That strategy resulted in recovery of 78 CNVs from the positive test set (two false negatives came from sample with tumour content < 20%, two had low level in FISH test, two came from low quality sample). All but one negative findings for CNVs were confirmed by WGS

SVs: Positive percentage agreement, PPA = 18/18 = 100%

SVs: Positive predictive value, PPV = 18/22 = 82%

SVs: False Positive Rate, FPR = 4/120 = 3%

CNVs: Positive percentage agreement, PPA = 78/86 = 91%

CNVs: Positive predictive value, PPV = 78/79 = 99%

CNVs: False Positive Rate, FPR = 1/30 = 3%

Note that this analysis assumes that FISH test is 100% sensitive and specific. This is unlikely to be true but making this assumption allows us to compare WGS results with standard of care. This validation exercise has covered only haematological samples and we anticipate that this behavior can be different for solid tumours. We are also planning to run similar validation on a cohort of sarcoma samples. Current analysis strategy involves subjective manual review of genome coverage and was conducted with FISH test results open for reviewer. We are planning to repeat this exercise with blind examination of reports by the team with diverse backgrounds (e.g. bioinformatician, haematologist, clinical scientist)

1.3 Overall summary of performance

Test	Metric	Value	Observed results
somatic small variants vs standard of care NGS panels	Positive percentage agreement	98.7%	155/157
	Positive predictive value	99.4%	155/156
somatic small variants vs high confidence exome data	Sensitivity, SNVs VAF>10%	98%	7832/7986
	Sensitivity, indels VAF>10%	95%	306/323
	Specificity, SNVs VAF>10%	90%	3298/3666
	Specificity, indels VAF>10%	85%	155/180
somatic structural variants vs standard of care FISH test	Positive percentage agreement	100%	18/18
	Positive predictive value	82%	18/22
	False Positive Rate	3%	4/120
somatic copy number variants vs standard of care FISH test	Positive percentage agreement	91%	78/86
	Positive predictive value	99%	78/79
	False Positive Rate	3%	1/30

2 Methods

2.1 Validation Sets

We have split the validation into different experiments depending on the category of variants in order to validate components and functionalities

Experiment 1: WGS somatic small variants calling against NGS panel standard of care tests (96 patients)

Experiment 2: WGS somatic small variants calling against high depth exome sequencing (16 genomes)

Experiment 3: WGS somatic CNVs/SVs calling against FISH standard of care tests (34 patients)

2.2 Measurements

Metrics	Description	Formula
Positive Percentage Agreement, PPA or Sensitivity	Fraction of relevant instances that have been retrieved over the total amount of relevant instances	number of True Positives/(number of True Positives + number of False Negatives)
Positive Predictive Value, PPV or Precision	Fraction of relevant instances among the retrieved instances	number of True Positives/(number of True Positives + number of False Positives)
False Positive Rate, FPR	Probability of false alarm	number of False Positives/(number of True Negatives + number of False Positives)

2.3 Output

All runs have been performed in production environment and results can be found on Interpretation portal.

3 Validation Experiment 1: Calling Somatic Small Variants

3.1 Background / Justification

In this experiment we estimate accuracy of calling, annotation and interpretation of somatic small variants using results of NGS panels standard of care tests as a truth set. 96 patients were selected based on availability of test data at GMCs.

3.2 Expected Outcome of Validation

We expect the following sensitivity for small variant calling: 95% of somatic SNVs with an allele frequency of ≥0.1 and 60% of somatic indels with an allele frequency of ≥0.2 (estimate is provided by Illumina and is based upon admixtures analysis of a highly accurate catalog of variants produced in the 'platinum genomes' project).

3.3 Methods

3.3.1 Method Overview

We collected results of panel testing that had been run locally by clinical laboratories in three GMCs and compared them with the WGS results. Cross-comparison of results from standard-of-care panels with WGS analysis allows us to estimate **sensitivity** and **precision** of Genomics England pipeline

3.3.2 Truth set sources

Details of panel testing:

- GMC1 - Qiagen Human Clinically Relevant Tumor Panel, Product no. 181900; Cat no. NGHS-101X. It is a UKAS iso-15189 accredited test in the lab. This panel contains 24 genes. Synonymous variants and variants observed in germline WGS are removed from the analysis. Panel analysis was done on an aliquot from the same FF DNA extraction sent for WGS.
- GMC2 - Oncomine Focuse assay. This assay was not intended as clinical essay. This panel contains 52 genes, validation data was returned for six genes that are relevant for the clinical management of colorectal cancer patients: BRAF, BRCA1, ERBB3, KRAS, NRAS and PIK3CA. Variants outside of hot spots list and variants observed in germline WGS are removed from the analysis. Panel analysis was done on an aliquot from the same FF DNA extraction sent for WGS.
- GMC3 – Ion Ampliseq hot spot cancer panel. It is a UKAS iso-15189 accredited test in the lab This panel contains 50 genes, validation data was returned for nine genes that are relevant for clinical management of colorectal and lung cancer patients: TP53, PTEN, PIK3CA, PDGFRA, KRAS, NRAS, KIT, EGFR, BRAF. Variants outside of hot spots list and variants observed in germline WGS are removed from the analysis. Panel analysis was done on an aliquot from FFPE DNA extraction different from the one sent for WGS. In case of discrepancies reported in the previous validation exercise testing was repeated with FF DNA sample

3.4 Validation Results

- **GMC 1 data set** - 15 patients with 28 somatic non-synonymous variants reported from panel testing (25 SNVs and 3 indels). WGS analysis missed to report 2/28 variants due to the low VAF in WGS data (3% and 4%). No additional non-synonymous variants were reported by WGS analysis in the regions overlapping with regions covered by panel
- **GMC 2 data set** - 22 patients with 23 somatic non-synonymous variants reported from panel testing (all SNVs). WGS analysis reported all variants. Additional PIK3CA variant came up in one patient with low VAF of 5%.
- **GMC 3 data set** - 59 patients with 105 somatic non-synonymous variants reported from panel testing (104 SNVs and 1 indel). WGS analysis reported 100 variants from this test set. Five missed variants (PTEN c.536G>A with 25% VAF in panel test, TP53 c.514G>T with 7% VAF, TP53 c.916C>T with 42% VAF, TP53 c.413C>T with 31% VAF, c.396G>T with 69% VAF) were manually reviewed and no evidence of the variant support was found in WGS data. We also confirmed good coverage for these positions (85-113 reads). Three additional variants came up in WGS analysis with VAFs in the range of 4-17%: TP53 c.742C>T at 14%, PIK3CA c.1624G>A at 4% and TP53 c.535C>G at 17%. We followed up with GMC on discrepant variants and the NGS panel testing was repeated with the same DNA prep extracted from FF tissue that was submitted for WGS. These new results were completely concordant with WGA findings. We assume that discrepant results are due to the tumour heterogeneity.

3.5 Conclusions

WGS data for 96 patients was validated against standard of care small panels. Based on the results of this experiment we can conclude that sensitivity of our pipeline for calling somatic small SNVs in clinically relevant genes is > 98%. Therefore, the results of our validation has exceeded estimate that was provided by Illumina and we consider this part of the pipeline is validated.

4 Validation Experiment 2: Calling Somatic Small Variants

4.1 Background / Justification

In this experiment we estimate accuracy of calling, annotation and interpretation of somatic small variants using results of high depth exome sequencing as a truth set. We also explore the sources of False Positive and False Negative for future improvements of our pipeline.

4.2 Expected Outcome of Validation

Same as in the experiment 1.

4.3 Methods

4.3.1 Method Overview

We were using high confidence data set from TRACERx study[4] (exome sequencing) to estimate **sensitivity** and **precision** of variant calling from WGS in Genomics England pipeline.

4.3.2 Truth set sources.

This data set was generated by the TRACERx consortium from high depth exome sequencing data (Agilent Human All Exome V5 capture, ~400x for each tumour and normal). The exome sequencing had previously been validated for a subset of variants with multiplex PCR (792 predicted non-silent mutations) and AmpliSeqTM custom panel (535 non-silent and 538 silent mutations). Their resulting sensitivity and precision estimates were > 99% (with exception of indel sensitivity estimate of 97%). We concluded that the highly accurate TRACERx exome data set is suitable for estimating accuracy of variant calling from WGS.

DNA for 16 TRACERx patients (tumour and matching normal) was subjected to WGS and outcomes of Genomics England pipeline were compared with exome data set.

VCF file with high confidence exome data set, was recovered from TRACERx publication Supplementary Table 3, and converted to a VCF using a custom script. Positions were lifted to GRch38. Exome and WGS VCF files were filtered to only contain exome positions using a BED-file for the custom genome coverage of TRACERx project, and WGS VCFs was converted to a modified VCF with additional information on the read depth (DP), variant frequency (FQ) and quality (QU), allowing us to compare genotype calls, VAF and whether the variant would have passed TRACERx and/or our filters.

For measurement of uncertainty downsampling for a TRACERx sample was repeated six times using several different random seeds; 578, 664, 752, 921, 988 and 99; downsampling levels - 70x, 80x, 90x, 100x, 110x and 120x

4.4 Validation Results

There was overall strong correlation for VAF recorded by TRACERx exomes and WGS (n=10,468, R2=0.88, p-value: < 2.2e-16). The correlation is maintained uniformly along frequency variants of different magnitude.

Figure below is showing comparison for VAFs between exome and WGS. Thick dashed lines are indicating VAF +/-5%, thin dashed lines are indicating VAF +/-10%, purple dots are indicating variants with VAF<10% in TRACERx exomes, green dots are indicating WGS False

Negatives with TRACERx VAF>10%, and grey dots are indicating WGS False Negatives with TRACERx VAF<10%.

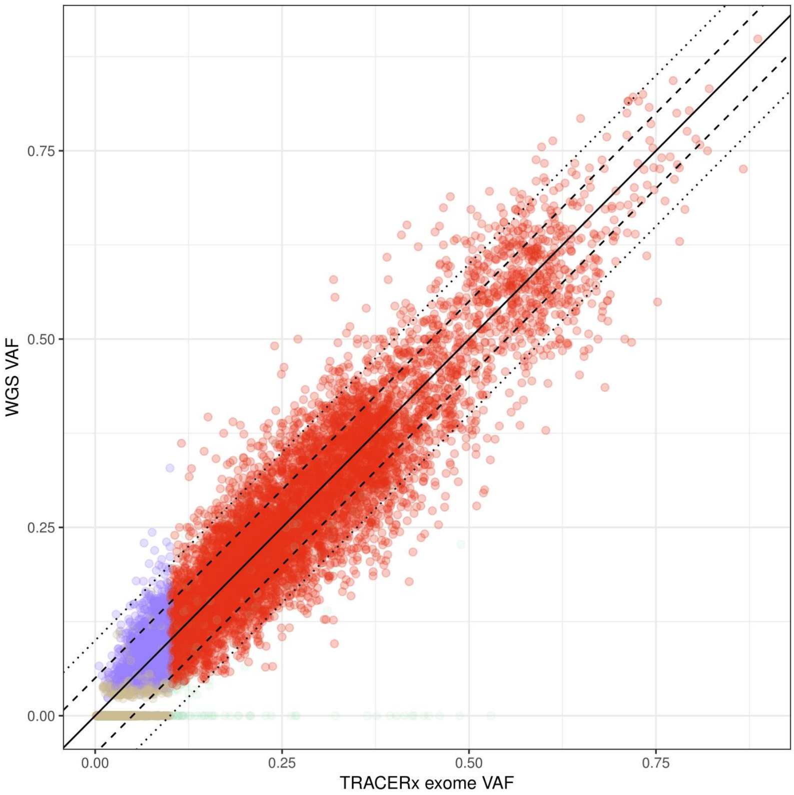

Overall, the VAFs estimated by TRACERx exomes and by WGS were very comparable, with 94% of WGS variants having VAFs within +/- 10% of TRACERx exome reported VAFs.

4.4.1 Sensitivity

For the 16 samples, the sensitivity for somatic SNVs with a VAF>10% (as estimated in exome sequencing) was 98% (sample range 93%-100%) from 7986 investigated variant positions. Sensitivity for somatic indels with a VAF>10% (as estimated in exome sequencing) over all 16 samples was 95% (80%-100% per sample) from 323 investigated variant positions. Matching indels identified by two different variant callers can be affected by different and equally valid ways to encode an indel as a variant. In order to ascertain that our statistics was not unduly influenced by such instances where the exact substitution was in agreement, but encoded differently by different software, we manually went through and curated all 8 instances of overlapping but un-merged variants between exome and WGS and corrected those which genuinely were in agreement about the variant

We further analysed the reasons for missing variant in WGS analysis. For the 2% of SNVs that are missed by WGS (potential False Negatives), VAF (estimated from BAM) has a lower value in WGS than in exome sequencing (see plot below). If there is a natural variance in approximated VAF by +/- 10%, it is possible that the False Negatives we have isolated are enriched in variants which by chance have a higher VAF in exome than in WGS. Some variants were detected by Strelka (blue, n=112) but rejected by the "LowQscore" filter. Others were not retrieved at all (green, n=33).

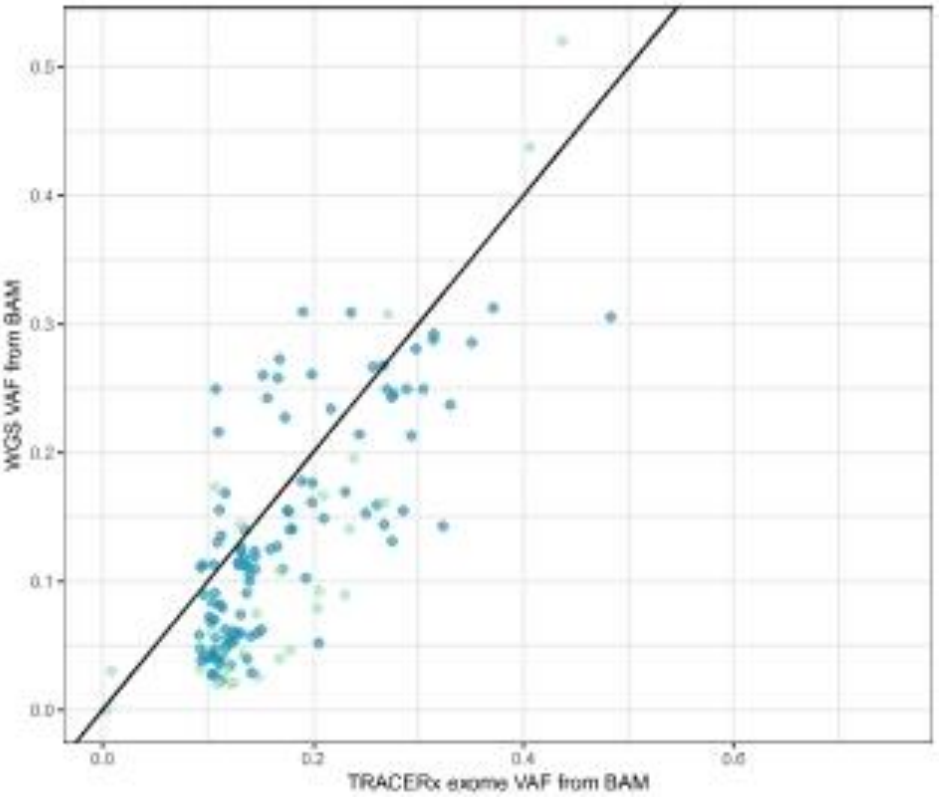

We noticed that VSQR-scores for variants missed by WGS are usually close to the 2.75 threshold (>2). However, lowering the threshold even to 2.5 would "rescue" approximately half of False Negative variants, while also approximately doubling the total number of called variants. We conclude that the 2.75 threshold is probably fairly well calibrated to achieve high sensitivity and avoid lowering precision. Out of the 33 SNVs not retrieved at all from WGS, we noticed that 4 had been mapped to an alt-allelle during the lift to GRCh38 (Illumina pipeline is not alt-aware). 19 variants had a VAF (calculated from the BAM) <10%. The remaining 10 suffer from poor overall mapping quality in the region.

There are 17 indels in the exome test set that are not called by WGS. Of the 17, only 3 were called by Strelka with similar VAF, but discarded with the "QSI_ref" filter. All 17 were manually reviewed, and the conclusion was that 5 should have been called, others are either indels in homopolymer run (8), have no support in WGS (2) or in the ambiguously mapped regions (2)

4.4.2 Precision

We also attempted to estimate precision using the exome dataset (fraction of variants that were reported in WGS analysis but were not supported by exome data set) although we anticipate that due to extensive filtering of potential False Positives in the exome data set it can miss True Positive variants and therefore precision can be under-estimated. Our calculations included only non-synonymous variants in protein coding genes (somatic variants in Domains 1-3 in WGA results). This subsetting removes a large proportion of variants of unknown importance and consequence (66% of indels and 65% SNVs). We also excluded from this analysis categories of somatic variants that are flagged in WGA results as potential False Positives - 17% of indels and 3% of SNVs (panel of normals, recurrent somatic variants, indels in homopolymer runs and noisy regions, simple repeats; for details see Technical information document). Across all 16 samples, the precision for somatic SNVs in the test set with a VAF>10% (as estimated from WGS) was 90% (sample range 57%-96%) from 3666 investigated variant positions. Precision for somatic indels with a VAF>10% (as estimated in WGS) over all 16 samples was 85% (sample range 50%-96%) from 180 investigated variant positions. Significant variability in precision estimate for individual samples can be explained by the small number of variants per sample, especially for indels

We further investigated potential sources for False Positive findings. Majority of potential False Positive variants are well supported by the exome sequencing and therefor they don't seem to represent artefact of sequencing or alignment. The plot below shows support (calculated from BAM file) for 377 SNVs that are missed by exome sequencing. The colour is by exome sequencing Q60 read depth. In total, 26% of potential False Positive variants have read support below 5th percentile (as calculated from the set of True Positives), and 11% of potential False Positive variants have read support above 95th percentile (as calculated from the set of True Positives). Therefor we conclude that potential False Positive variants that are missed by exome sequencing are biased towards both lower and higher Q60 read depth. This indicates that some False Positives may appear in regions with mappability issues. That should not be very common in the exonic regions but is possible for instance in large gene families or rapidly evolving genes, where the reference genome may fail to accurately represent the patient genome. We randomly picked and reviewed 10 variants with low read depth and 10 variants with high read depth. Under visual assessment all these variants appeared accurately called in WGS.

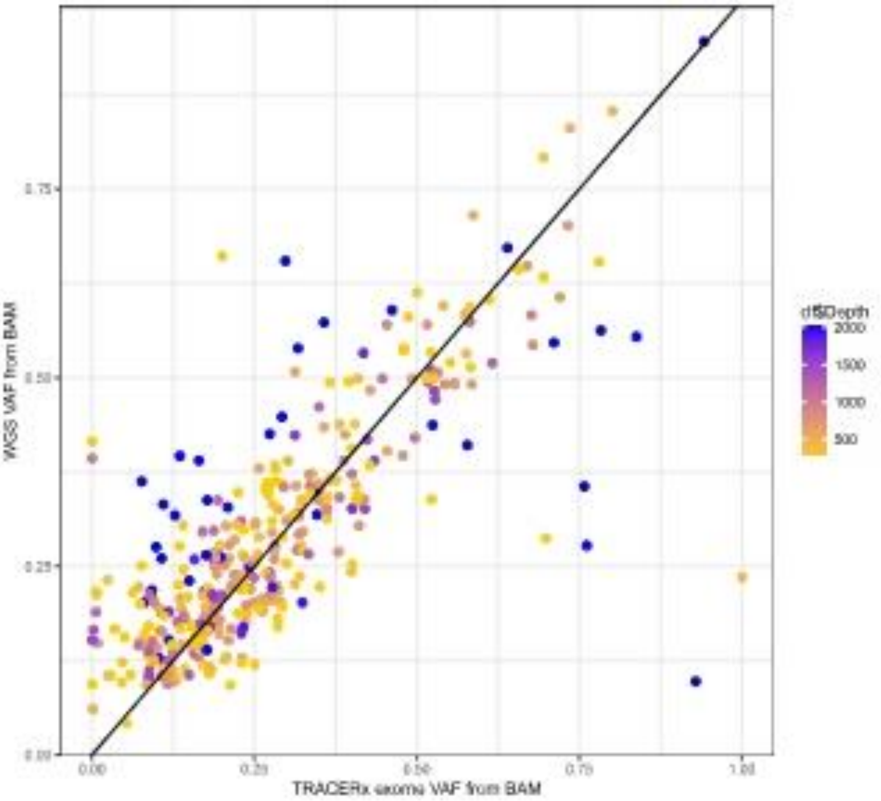

For 223 potential False Positive SNVs with coverage in the range between 5th and 95th percentile (as calculated from the set of True Positives), we randomly picked up and reviewed 20 variants. They are all well supported by read coverage on both strands, mapping well, absent from normal and VAF in tumour is similar to the reported. This suggests that a large proportion of the remaining unexplained False Positives are actually True Positives. Given that there is such a strong correlation between VAFs from exome data and WGS (adjusted R2=0.60, p< 2.2e-16), we believe a large set of these False Positives may indeed be real variants that were filtered with the blacklist of genomic regions used by TRACERx project (regions with mappability issues excluded from the Encode project, simple repeats, segmental duplications and microsatellite regions). Blacklisted regions are very unlikely to overlap with clinically-relevant genes from Domains 1 and 2 but can contribute to mutation burden and signatures calculation.

We further reviewed 25 potential False Positive indels and they all look true in WGS. 11 of these indels failed to be discovered from exome sequencing data due to poor support, low depth or poor mapping quality.

4.4.3 Measurement of uncertainty

We decided to address uncertainty of our test and its responsivity to WGS coverage depth by evaluating how the number of consistently recovered variants changes as a function of coverage and by repeatedly sampling reads from a sample that has higher coverage. Reads from WGS for the same sample were downsampled to 6 different depths (70x, 80x, 90x, 100x, 110x and 120x), 6 times for each depth using different random seeds. 84% of all variants were recovered every time for 70x sample, while for 120x this value rises to 93%. At 100x, which is our normal delivery depth, reproducibility for recovering all variants was 92%, while for variants with VAF>10% it raises to 98%.

Figure below shows percentage of variants that are consistently recovered when the sample is downsampled six times to the same depth with different seed (variants with VAF>10% in light blue)

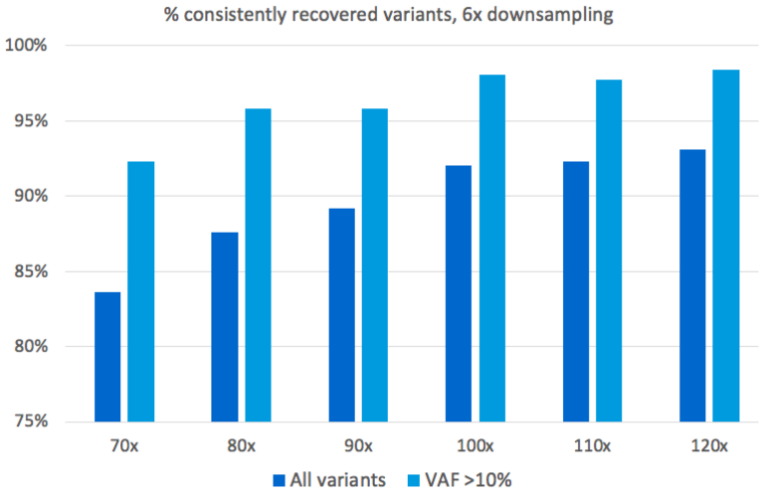

As expected the level of variation will decrease as the sequencing depth increases and variants with lower VAF will be most affected by stochastic events. Variants with VAF>10% are reliably re-recovered with 100x sequencing depth and further increase in coverage does not improve reproducibility.

4.5 Limitations

All samples in this experiment underwent PCR-free library preparation and cross-patient contamination in tumour samples was < 1%. Therefore this validation does not cover samples that underwent Nano sequencing library preparation protocol or samples with 1-5% of contamination (those samples are flagged in WGA results sent to GMC; results for samples with > 5% contamination are not returned to GMC and replacing samples are requested by Sequencing team). Level of cross-sample contamination and sequencing library preparation method are clearly flagged in the WGA results.

Our experiment for measurement of uncertainty was limited to the variant calling step. We are working on proposal together with Illumina to estimate reproducibility of end-to-end pipeline from re-sequence genomes for cohort of 10 patients.

4.6 Conclusions

To summarise, we performed WGS assay validation using samples of the type intended for the assay (DNA extracted from fresh tissue) with a variety of tumour purities so that test performance is representative of the larger population. WGS data was validated against whole exome data with multiple genes and variety of variant allele frequencies covered.

Based on the results of this experiment we can conclude that sensitivity of our pipeline for somatic small SNVs with a VAF >10% is > 98% and for indels > 95%. Therefore the results of our validation has exceeded estimate that was provided by Illumina and we consider this part of the pipeline is validated.

In addition we estimated uncertainty of our test. At 100x coverage reproducibility of recovering variants with VAF>10% achieves 98% and does not benefit from higher depth

5 Validation Experiment 3: Calling Somatic Copy Number Variants and Structural Variants

5.1 Background / Justification

In this experiment we calculate accuracy of calling, annotation, interpretation and visualisation of somatic CNVs and SVs using results of FISH standard of care tests as a truth set. 35 patients were selected based on availability of test data at GMCs.

5.2 Expected Outcome of Validation

Existing test sets for SVs/CNVs calling are very limited. From validation presented by Munich Leukaemia Lab (personal communication): WGS identified 94% (98/104) of translocations reported by FISH and 72% (71/98) of CNV regions identified by cytobanding analysis. MLL is using the same algorithm as our pipeline for SV detection (Manta) and GATK algorithm for CNV detection. This pipeline does not relay on matching germline and therefor it struggles from high rate of false positives (numbers are not reported)

5.3 Methods

5.3.1 Method Overview

We collected results of CNV/SV testing that had been run locally by clinical laboratories in three Genomic Medicine Centres and compared them with the WGS results. CNV analysis combined Canvas calls with visual observations of coverage in specific genomic regions on circos plot

5.3.2 Truth set sources

Details of FISH testing:

- GMC1 - this set contains 22 patients with paediatric ALL. This set contains broad variety of SVs/CNVs in genes that are relevant for clinical management of ALL patients (ABL2, BCR-ABL1, BCL6, CDKN2A, CSF1R, ETV6-RUNX1, IGH-MYC, IGH-BCL2, HLF, KMT2A, MECOM, MYC, PDGFRB, STIL-TAL1, TLX1, TLX3, TCL1, TCF3-PBX1, TRG, TRB, TRAD) as well as centromeric probes for detecting high hyperdiploid karyotype
- GMC2 – this set contains 11 patients with AML, CLL, multiple myeloma, lymphoma. This set contains IGH translocations as well as Gains/Losses of chromosomes 1 and 17 and inversion on chromosome 16
- GMC3 - this is a small set of only two patients with multiple myeloma. This set contains IGH translocations as well as Gains/Losses of chromosomes 1 and 17

5.4 Validation Results

Positive: present in sample according to test

Negative: absent in sample according to test

- **GMC 1 data set -** 11 patients with 25 somatic SVs (5 positives and 20 negatives) and 27 CNVs (7 positives and 20 negatives) reported by FISH testing. WGS was calling one negative CNV finding

- **GMC 2 data set** - two patients with 5 somatic SVs (1 positive and 4 negatives) and 7 CNVs (5 positives and 2 negatives) reported by FISH testing. WGS was missing three positive CNV findings: one was reported by FISH at low level (8%) and two came from low quality sample (Nano library prep with high indels burden)
- **GMC 3 data set** - 22 patients with 108 somatic SVs (12 positive and 96 negatives) and 82 CNVs (74 positives and 8 negatives) reported by FISH testing. WGS identified four translocations that were reported as negative findings by FISH test. We inspected support for these translocation in IGV viewer and report reciprocal translocations with very strong support for all four SVs. GMC is currently repeating FISH testing for this rearrangements. WGS missed five positive CNV findings (one of them was detected by FISH with low level (14.5%) and one came from the sample with tumour content < 20%).
- **Confusion matrix for CNV validation over three GMCs**

	FISH positive	FISH negative	
**WGS positive**	True positive<br>78 variants	False positive<br>1 variants	79
**WGS negative**	False negative<br>8 variants	True negative<br>29 variants	37
	86	30	116

Positive percentage agreement, PPA = 78/86 = 91%
Positive predictive value, PPV = 78/79 = 99%
False Positive Rate, FPR = 1/30 = 3%

- **Confusion matrix for SV validation over three GMCs**

	FISH positive	FISH negative	
**WGS positive**	True positive<br>18 variants	False positive<br>4 variants	22
**WGS negative**	False negative<br>0 variants	True negative<br>116 patients	116
	18	120	138

Positive percentage agreement PPA = 18/18 = 100%
Positive predictive value PPV = 18/22 = 82%
False Positive Rate, FPR = 4/120 = 3%

5.5 Limitations

This validation exercise has covered only haematological samples and we anticipate that this behaviour can be different for solid tumours. We are also planning to run similar validation on a cohort of sarcoma samples.

Current analysis strategy involves subjective manual review of genome coverage and was conducted with FISH test results open for reviewer. We are planning to repeat this exercise with blind examination of reports by the team with diverse backgrounds (e.g. bioinformatician, haematologist, clinical scientist).

We also started benchmarking alternative CNV callers that would allow to detect true CNV variants without additional review of coverage.

5.6 Conclusions

Comparison of WGS with the results of FISH test demonstrated Positive Percentage Agreement > 90% for both SVs and CNVs and False Positive Rate < 5%. These values exceeded our expectations.

6 List of Abbreviations

Abbreviation	Explanation
GMC	Genomic Medicine Center
WGS	Whole Genome Sequencing
WGA	Whole Genome Analysis
CNV	Copy Number Variant
SV	Structural Variant
VAF	Variant Allele Frequency
SNV	Single Nucleotide Variant
Indel	Insertion or Deletion
VCF	Variant Call Format
FFPE	Formalin-Fixed Paraffin-Embedded
FISH	Fluorescence in situ hybridization

7 References

[1] Guidelines for Validation of Next-Generation Sequencing-Based Oncology Panels: A Joint Consensus Recommendation of the Association for Molecular Pathology and College of American Pathologists. *Jennings LJ et al.* J Mol Diagn. 2017; 19:341-365

[2] Isaac: ultra-fast whole-genome secondary analysis on Illumina sequencing platforms. *Raczy et al.* Bioinformatics. 2013; 29:2041-2043

[3] Strelka: accurate somatic small-variant calling from sequenced tumor-normal sample pairs. *Saunders et al.* Bioinformatics. 2012; 28:1811-1817

[4] Tracking the Evolution of Non–Small-Cell Lung Cancer. *Jamal-Hanjani M et al.* N Engl J Med 2017; 376:2109-2121
