# Supplementary material for: Insights for precision oncology from the integration of genomic and clinical data of 13,880 tumors from the 100,000 Genomes Cancer Programme
Source: Nat Med. 2024 Jan 11;30(1):279–89. doi: 10.1038/s41591-023-02682-0 (PMC10803271; doi:10.1038/s41591-023-02682-0)
Supplement: Supplementary file 2 — Reporting Summary [file 41591_2023_2682_MOESM2_ESM.pdf]

nature portfolio

Corresponding author(s): Mark Caulfield
Nirupa Murugaesu

Last updated by author(s): Oct 15,2023

Reporting Summary

Nature Portfolio wishes to improve the reproducibility of the work that we publish. This form provides structure for consistency and transparency in reporting. For further information on Nature Portfolio policies, see our Editorial Policies and the Editorial Policy Checklist.

Statistics

For all statistical analyses, confirm that the following items are present in the figure legend, table legend, main text, or Methods section.

n/a	Confirmed	
☐	☒	The exact sample size (*n*) for each experimental group/condition, given as a discrete number and unit of measurement
☒	☐	A statement on whether measurements were taken from distinct samples or whether the same sample was measured repeatedly
☐	☒	The statistical test(s) used AND whether they are one- or two-sided<br>*Only common tests should be described solely by name; describe more complex techniques in the Methods section.*
☐	☒	A description of all covariates tested
☐	☒	A description of any assumptions or corrections, such as tests of normality and adjustment for multiple comparisons
☐	☒	A full description of the statistical parameters including central tendency (e.g. means) or other basic estimates (e.g. regression coefficient) AND variation (e.g. standard deviation) or associated estimates of uncertainty (e.g. confidence intervals)
☐	☒	For null hypothesis testing, the test statistic (e.g. *F*, *t*, *r*) with confidence intervals, effect sizes, degrees of freedom and *P* value noted<br>*Give P values as exact values whenever suitable.*
☒	☐	For Bayesian analysis, information on the choice of priors and Markov chain Monte Carlo settings
☒	☐	For hierarchical and complex designs, identification of the appropriate level for tests and full reporting of outcomes
☒	☐	Estimates of effect sizes (e.g. Cohen's *d*, Pearson's *r*), indicating how they were calculated

Our web collection on statistics for biologists contains articles on many of the points above.

Software and code

Policy information about availability of computer code

Data collection

OpenClinica v3.4

Data analysis

SEquencing data QC, Mapping and variant calling
North Star pipeline version 2.6.53.23
ISAAC version iSAAC-03.16.02.19
Starling version 2.4.7
Strelka version 2.4.7
Canvas version 1.3.1
Manta version 0.28.0
samtools version 1.9

R packages used in this analysis
R4.0.3
survminer_0.4.9
survival_3.2-7
dplyr_1.0.7
purrr_0.3.4
tidyr_1.1.2
tibble_3.0.3
ggplot2_3.3.2
tidyverse_1.3.0

Rlabkey_2.7.0
RColorBrewer_1.1-2

The code is available on GitLab (https://gitlab.com/genomicsengland/genomics_england_publications/100k_cancer_programme/) and has been uploaded to https://doi.org/10.5281/zenodo.8311292.

For manuscripts utilizing custom algorithms or software that are central to the research but not yet described in published literature, software must be made available to editors and reviewers. We strongly encourage code deposition in a community repository (e.g. GitHub). See the Nature Portfolio guidelines for submitting code & software for further information.

Data

Policy information about availability of data

All manuscripts must include a data availability statement. This statement should provide the following information, where applicable:

- Accession codes, unique identifiers, or web links for publicly available datasets
- A description of any restrictions on data availability
- For clinical datasets or third party data, please ensure that the statement adheres to our policy

Public datasets that were used for variant annotation:
Ensembl version 90/GRCh38
COSMIC version v86
ClinVar October 2018 release
COSMIC signatures v3

The data supporting the findings of this study are available within the Research Environment, a secure cloud workspace. Details on how to access data for this publication can be found at https://re-docs.genomicsengland.co.uk/pan_cancer_pub/. Additional processed aggregated data used to generate figures can be found in Supplementary Tables S5-S20.

To access genomic and clinical data within this Research Environment, researchers must first apply to become a member of either the Genomics England Clinical Interpretation Partnership, GECIP (https://www.genomicsengland.co.uk/research/academic) or the Discovery Forum (industry partners https://www.genomicsengland.co.uk/research/research-environment). The process for joining the GECIP is described at https://www.genomicsengland.co.uk/research/academic/join-gecip and consists of the following steps:
1. Your institution will need to sign a participation agreement available at https://files.genomicsengland.co.uk/documents/Genomics-England-GeCIP-Participation-Agreement-v2.0.pdfand email the signed version to gecip-help@genomicsengland.co.uk.
2. Once you have confirmed your institution is registered and have found a GECIP domain of interest, you can apply through the online form at https://www.genomicsengland.co.uk/research/academic/join-gecip. Once your Research Portal account is created you will be able to log in and track your application.
3. The domain lead will review your application within 10 working days.
4. Your institution will validate your affiliation.
5. You will complete our online Information Governance training and will be granted access to the Research Environment within 2 hours of passing the online training.

Data that has been made available to registered users include: alignments in BAM or CRAM format, annotated variant calls in VCF format, signatures assignment, tumour mutation burden, sequencing quality metrics, summary of findings that is shared with Genomic Lab Hubs, secondary clinical data as described in this paper. Further details of the types of data available (for example, mortality, hospital episode statistics and treatment data) can be found at https://re-docs.genomicsengland.co.uk/data_overview/. Germline variants can be explored in Interactive Variant Analysis Browser (see description at https://re-docs.genomicsengland.co.uk/iva_variant/). Cancer patients cohort and longitudinal clinical information on treatment and mortality can be explored with Participant Explorer (see description at https://re-docs.genomicsengland.co.uk/pxa/).

Research involving human participants, their data, or biological material

Policy information about studies with human participants or human data. See also policy information about sex, gender (identity/presentation), and sexual orientation and race, ethnicity and racism.

Reporting on sex and gender	Biological sex that was inferred using the ratio of mean sequencing coverage of sex chromosomes and mean sequencing coverage of autosomes. In our analysis, patients were not stratified by sex to maximize the power of the cohort.<br>Patients provided informed consent for paired tumour and normal (germline) whole genome sequencing (WGS) analysis. Participants also gave consent for their genomic data to be linked to anonymised longitudinal health records and shared with researchers in a secure Research Environment.
Reporting on race, ethnicity, or other socially relevant groupings	Socially relevant categorization variables were not used in this study.
Population characteristics	15,241 patients diagnosed with cancer within the NHS that were recruited to the Cancer Programme of the 100,000 Genomes Project between 2015 and 2019. Tumour types with more than 1,000 sequenced tumour genomes included breast invasive carcinoma (n=2925), colon adenocarcinoma (n=1948), sarcoma (n=1617) and kidney renal clear cell carcinoma (n=1163). 11.9% (1,645/13,880) of patients had stage 4 cancer (advanced metastatic disease). Early onset (median age <50 years) was observed for low grade glioma and testicular germ cell tumours in agreement with incidence statistics. Tumour samples mainly originated from surgical resections (94.5%, n=13,120), including 93.6% treatment-naive cases and 6.4% post-neoadjuvant treatment. Only 5.5% (n=760) came from metastatic or diagnostic biopsies, with 10.9% (n=83) being post-treatment.

Recruitment	Participants were selected on the basis of having been identified by health care professionals and researchers within the NHS as having a cancer diagnosis. The participants were recruited across 13 NHS Genomic Medicine Centres and written informed consent was obtained from the participants.
Ethics oversight	Research described in this manuscript complies with all relevant ethical regulations. Approval for the project was obtained from the East of England - Cambridge South Research Ethics Committee (REC reference 14/EE/1112, IRAS ID 166046)

Note that full information on the approval of the study protocol must also be provided in the manuscript.

Field-specific reporting

Please select the one below that is the best fit for your research. If you are not sure, read the appropriate sections before making your selection.

☒ Life sciences ☐ Behavioural & social sciences ☐ Ecological, evolutionary & environmental sciences

For a reference copy of the document with all sections, see nature.com/documents/nr-reporting-summary-flat.pdf

Life sciences study design

All studies must disclose on these points even when the disclosure is negative.

Sample size	The results shown here are not the result of an experimental set up. We are describing observations for a cohort of 13,880 cancer patients recruited for 100,000 Genomes Programm. Sample size calculation is not relevant for this study.
Data exclusions	Pediatric cancers, hematological malignancies, cancers of unknown primary ans samples that didn't have clinical information from secondary sources were excluded as stated in the manuscript.
Replication	Replication is not relevant for the reason explained above
Randomization	Randomization is not relevant for the reason explained above
Blinding	Blinding is not relevant for the reason explained above

Reporting for specific materials, systems and methods

We require information from authors about some types of materials, experimental systems and methods used in many studies. Here, indicate whether each material, system or method listed is relevant to your study. If you are not sure if a list item applies to your research, read the appropriate section before selecting a response.

Materials & experimental systems

n/a	Involved in the study	
☒	☐	Antibodies
☒	☐	Eukaryotic cell lines
☒	☐	Palaeontology and archaeology
☒	☐	Animals and other organisms
☒	☐	Clinical data
☒	☐	Dual use research of concern
☒	☐	Plants

Methods

n/a	Involved in the study	
☒	☐	ChIP-seq
☒	☐	Flow cytometry
☒	☐	MRI-based neuroimaging

Plants

Seed stocks	Not applicable
Novel plant genotypes	Not applicable
Authentication	Not applicable
